# Quality Assessment of Commercial Dry-Aged Beef Produced in Poland

**DOI:** 10.3390/foods15132345

**Published:** 2026-07-02

**Authors:** Marta Chmiel, Lech Adamczak, Marcin Bryła, Agata Żak-Kułakowicz, Elżbieta Hać-Szymańczuk, Tomasz Florowski, Danuta Jaworska, Wiesław Przybylski, Ewelina Zielińska, Krzysztof Mrozik, Dominik Popowski, Marek Roszko, Edyta Juszczuk-Kubiak

**Affiliations:** 1 Department of Food Technology and Assessment, Warsaw University of Life Sciences—SGGW (Szkoła Główna Gospodarstwa Wiejskiego w Warszawie), 159C Nowoursynowska Street, 02-776 Warsaw, Poland; lech_adamczak@sggw.edu.pl (L.A.); tomasz_florowski@sggw.edu.pl (T.F.); 2 Department of Food Safety and Chemical Analysis, Prof. Wacław Dąbrowski Institute of Agricultural and Food Biotechnology-State Research Institute, 36 Rakowiecka Street, 02-532 Warsaw, Poland; marcin.bryla@ibprs.pl (M.B.); agata.zak-kulakowicz@ibprs.pl (A.Ż.-K.); dominik.popowski@ibprs.pl (D.P.); marek.roszko@ibprs.pl (M.R.); 3 Department of Food Biotechnology and Food Microbiology, Warsaw University of Life Sciences—SGGW (Szkoła Główna Gospodarstwa Wiejskiego w Warszawie), 159C Nowoursynowska Street, 02-776 Warsaw, Poland; elzbieta_hac_szymanczuk@sggw.edu.pl; 4 Department of Food Gastronomy and Food Hygiene, Institute of Human Nutrition Sciences, Warsaw University of Life Sciences—SGGW (Szkoła Główna Gospodarstwa Wiejskiego w Warszawie), 159C Nowoursynowska Street, 02-776 Warsaw, Poland; wieslaw_przybylski@sggw.edu.pl; 5 Department of Biotechnology, Prof. Wacław Dąbrowski Institute of Agricultural and Food Biotechnology-State Research Institute, 36 Rakowiecka Street, 02-532 Warsaw, Poland; ewelina.zielinska@ibprs.pl (E.Z.); krzysztof.mrozik@ibprs.pl (K.M.); edyta.juszczuk-kubiak@ibprs.pl (E.J.-K.)

**Keywords:** dry-aged beef, crust, fungal community, sensory evaluation, quality

## Abstract

The aim of the study was to evaluate the physicochemical, microbiological, and biochemical quality of commercially available dry-aged beef produced in Poland, with particular emphasis on differences between the crust and the interior of steaks. Fourteen samples (11 rib-eye and 3 sirloin) from different producers were analysed. The colour parameters, pH, water activity, thiobarbituric acid reactive substances, water-holding capacity, basic chemical composition, texture, microbial counts, free amino acids, biogenic amines profiles, and consumer sensory quality were determined. Microbiological analyses covered total plate count, psychrotrophic bacteria, lactic acid bacteria, Enterobacteriaceae, *Pseudomonas* spp., *Brochothrix thermosphacta*, yeasts and Moulds, as well as the presence of *Salmonella* spp. and *Listeria monocytogenes*. Fungal species isolated from selected crust samples were identified using ITS rDNA sequencing. Significant variability among products was observed in colour (L*: 31.1–45.0), pH (5.3–6.7), and water activity (0.92–0.99). Compared with the interior, the crust exhibited higher lipid oxidation (TBARS up to 2.31 mg MDA/kg), higher microbial loads, and greater accumulation of biogenic amines, including tyramine, cadaverine, and putrescine. Rib-eye 6, Rib-eye 7, Rib-eye 8, Rib-eye 10, and Rib-eye 11 showed the highest sensory acceptance. On the crust of selected highly rated steaks filamentous fungi such as *Mucor flavus* and *Thamnidium elegans* were identified, which suggests environmental colonisation and potential technological relevance.

## 1. Introduction

In 2023, the average beef consumption in Poland was approximately 0.8 kg per capita, a significant decrease from previous years, positioning beef as the least-consumed meat, far behind pork and poultry. In 2024, beef consumption increased to 3.3 kg per capita [1]. Although beef consumption in Poland remains relatively low, dry-aged beef is gaining visibility as a premium product, in line with the broader global increase in interest in high-quality beef with distinctive sensory characteristics. This trend is associated with growing consumer demand for superior flavour and texture, as well as unique culinary experiences. The popularity of dry-aged beef is further reinforced by restaurants and steakhouses aiming to distinguish their menus with exclusive steak offerings. Due to its unique sensory properties and premium market positioning, dry-aged beef can achieve higher prices, thereby increasing its commercial attractiveness [2,3,4,5]. Dry-aged beef is typically regarded as a niche and exclusive product [6,7]. The dry-ageing process improves beef palatability by increasing tenderness and developing desirable mushroom-like, nutty, or buttery flavours [8,9,10,11]. The meat is aged, unpacked, in dedicated ageing chambers under controlled conditions of temperature, relative humidity, and air velocity, typically for a minimum of 14 days, although longer ageing periods of several weeks could also be applied [12,13,14].

During ageing, moisture evaporates from the meat’s surface layers, forming a dry crust. This crust is usually trimmed and discarded before sale. On the crust, a specific surface microbiota growth is observed, including moulds such as *Penicillium* (e.g., *P. nalgiovense*), *Mucor* (e.g., *M. flavus*), and *Thamnidium* (e.g., *T. elegans*), along with yeasts such as *Debaryomyces hansenii* [3,4,10,15,16]. The microorganisms release extracellular proteases and lipases, which contribute to meat tenderization and lipid oxidation, thereby enhancing the formation of branched aldehydes, methyl ketones, and esters that define the characteristic dry-aged beef flavour profile [10,17,18]. In most cases, microbiota development is spontaneous and difficult to control. However, recent studies explored controlled inoculation using microbial biostarters, demonstrating that selected moulds and yeasts can influence ageing outcomes. For example, *Penicillium candidum* or *Penicillium nalgiovense* biostarters accelerate the development of desirable flavour and improve sensory characteristics. Moreover, *Mucor flavus* biostarters modulate volatile compound profiles, texture parameters (hardness), and overall acceptability after 21–28 days of ageing [19,20].

The growing popularity of dry-aged beef has prompted meat processing plants, retailers, butcher shops, and restaurants to produce dry-aged beef in-house. Some of these are using commercially available small-scale ageing chambers [6]. However, the use of such equipment, particularly by non-professional food operators, raises microbiological safety concerns, an issue that the European Food Safety Authority (EFSA) has recently addressed [21].

According to the EFSA [21], when operated under hygienic conditions, including proper refrigeration, filtered airflow, and regular sanitation, typical dry-ageing does not inherently increase the risk of pathogenic contamination compared with conventional wet-ageing. Nevertheless, potential hazards are concentrated in the crust, where moulds proliferate. Therefore, key control points include maintaining hygiene standards, proper trimming of the crust, and preventing and monitoring toxigenic fungi and their metabolites (e.g., mycotoxins). Regular verification of parameters set up in ageing chambers is also essential to ensure product safety [22]. Studies investigating the quality of commercial dry-aged beef, including associated microbiota, have been conducted mainly in Asian countries [10,15,23], with only a few exceptions, such as the studies of Gowda et al. [4] in Belgium, Ribeiro et al. [24] in Portugal, and Coton et al. [22] in France. Despite the growing interest in dry-aged beef, comprehensive assessments of commercially available products in Europe remain scarce, and data from central and eastern Europe are particularly limited. Moreover, commercial dry-ageing is inherently characterised by substantial variability in raw materials, processing conditions, and handling practices, which may result in pronounced differences in product quality, including microbiological, and safety-related indicators, especially within the crust layer that is routinely removed before consumption.

Therefore, this study aimed to characterise the physicochemical, textural, and microbiological quality of commercially available dry-aged beef produced in Poland, to compare key quality traits between the crust and the interior of steaks, and to identify fungal communities associated with steaks exhibiting the highest sensory scores. To the authors’ knowledge, this is the first comprehensive study to address these aspects of dry-aged beef produced in Poland.

## 2. Materials and Methods

### 2.1. Study Design and Sample Preparation

To evaluate the quality of dry-aged beef available on the Polish market, raw material was obtained from 14 domestic companies specialising in this ageing method, in three independent experimental series. From each company, a set of 12 beef slices (2 cm thick) originating from a single dry-aged primal cut (rib-eye or sirloin) was purchased. The steaks were prepared by the producers. The first slice from each dry-aged primal cut was discarded because it consisted predominantly of the heavily dehydrated end surface formed during ageing. Subsequently, twelve steaks, approximately 2 cm thick, were cut from the freshly exposed surface using a band saw. The dried surface layer (crust) located on the outer perimeter of each steak was left intact and used for subsequent analyses. The producers vacuum-packed the steaks prior to delivery and transported them to the laboratory under refrigerated conditions. All analyses were initiated immediately upon receipt of the samples to minimize additional storage time before examination. The beef had been dry-aged for at least 21 days under controlled conditions (temperature, relative humidity, and air velocity) in an ageing chambers or cabinets. As the study was designed as an observational market survey, detailed information regarding animal-related factors, including breed, age, sex, carcass weight, feeding system, and production system, was not available. Similarly, the producers did not disclose complete and detailed dry ageing parameters. Therefore, the observed differences among samples are interpreted as reflecting the combined effect of raw material variability and producer-specific dry ageing practices.

From each set of twelve slices, one slice was subjected to physicochemical quality analysis, which included measurements of colour parameters (L*, a* and b*), water activity (aw), pH, thiobarbituric acid reactive substances (TBARS) index value, water-holding capacity (WHC), and the basic chemical composition (water, protein, and fat content). The second slice was used to determine cooking yield and then to measure shear force and texture profile analysis (TPA). Another two steaks were used to determine the profiles of free amino acids, biogenic amines, and fatty acids. The next slice was allocated for microbiological quality evaluation, and two slices for mould identification. The remaining five slices were used for sensory evaluation. All analyses (except for colour parameters, cooking yield, and texture measurements) were conducted after dividing each slice into two parts: the crust (trimmings), i.e., the dried outer layer from the perimeter of the steak, and the interior, defined as the edible muscle tissue remaining after removal of the crust from the steak perimeter. The crust and interior portions of each steak intended for the determination of pH, TBARS value, WHC, and basic chemical composition, as well as for the analysis of free amino acids, biogenic amines, and fatty acid profiles, were ground using an IKA MultiDrive (IKA, Warsaw, Poland) crusher (15,000 rpm, 10 s) equipped with an MT 150.5 stainless steel beater chamber. The preparation of steaks for sensory evaluation is described in a separate section.

### 2.2. Methods

#### 2.2.1. L*, a* and b* Colour Parameters

Colour parameters (L*, a*, and b*) were measured on the cut surface of beef steaks (the interior part of the steak, on meat tissue, taking caution to avoid large areas of connective tissue and marbling). Prior to measurement, steaks were removed from vacuum packaging and allowed to bloom for 30 min under atmospheric conditions. The measurement was performed in five repetitions, and the mean value was taken as the final result. A MINOLTA^®^ CR-400 colourimeter (Minolta, Tokyo, Japan) was used for the measurements. The following device settings were used for both calibration and measurement: D65 light source, 10° standard observer, and an 8 mm aperture. Prior to measurement, the device was calibrated using a white calibration plate (Y = 95.2, x = 0.3159, y = 0.3326).

#### 2.2.2. Water Activity

The water activity of the crust and interior of beef steaks was measured using an AquaLab CX-2 (Decagon Devices Inc., Pullman, WA, USA) at 25 ± 1.5 °C. For the crust measurement, 3 mm-thick slices were collected with a sterile scalpel, cut parallel to the steak surface to ensure only the outer layer was sampled, avoiding any internal meat portions. For the interior measurements, samples were taken from the central part of the steak, approximately 4 cm beneath the surface, with care taken to avoid large areas of connective tissue and marbling. Water activity was measured in triplicate, and the mean value was used as the final result.

#### 2.2.3. pH Level

The pH of the crust and the interior of steaks was measured using a Testo 206 pH meter (Testo SE & Co. KGaA, Titisee-Neustadt, Germany). The electrode and temperature probe were inserted directly into the grounded crust or interior part of each steak. Before use, the pH meter was calibrated with standard buffer solutions at pH 4.0 and 7.0. Measurements were performed in triplicate for each ground crust or interior portion, and the mean values were used as the final results.

#### 2.2.4. TBARS Index

The TBARS index values of the crust and interior of each steak were determined using the method described by Florowski et al. [25]. The blank sample consisted of 5 mL of 2-thiobarbituric acid (TBA) solution and 5 mL of 10% trichloroacetic acid (TCA) (Sigma-Aldrich Sp. z o.o., Poznań, Poland). The samples were incubated in a boiling water bath for 35 min to allow colour development before absorbance measurement. After incubation, the samples were immediately cooled. Absorbance was measured at 532 nm with a CampSpec spectrophotometer (CampSpec Ltd., Leeds, UK).

The TBARS were expressed as malondialdehyde (MDA) equivalents (mg MDA/kg of product) using a standard curve with malonaldehydebis (dimethylacetal), the slope of which was 2.34. All measurements were performed in triplicate, and the mean value was used as the final result.

#### 2.2.5. Water-Holding Capacity (WHC)

The water-holding capacity of the crust and interior of steaks was determined using the filter-paper press method, as described by Grau and Hamm [26], modified by Pohja and Niinivaara [27] and used by Jaworska et al. [28]. In brief, approximately 0.3 g of meat was placed on Whatman No. 1 filter paper (preconditioned at saturated KCl solution for 24 h), sandwiched between two glass plates, and loaded with a weight (2 kg). After 5 min of compression, the pressed meat sample and the resulting exudate stain on the filter paper were outlined and scanned. The surface areas corresponding to the pressed meat and the exudate stain were determined using digital planimetry with ImageJ ver 1.53e software. The WHC results were expressed as cm^2^/g.

#### 2.2.6. Water, Protein, and Fat Content

The water, protein, and fat contents of the crust and interior part of steaks were determined according to Polish Standards. Moisture content was determined following PN-ISO 1442:2000 [29], by drying samples at 105 °C in an SUP-65 dryer (Wamed, Warsaw, Poland). Protein content was determined by the Kjeldahl method in accordance with PN-75/A-04018:2002 using a Velp Scientifica UDK 129 distillation unit (Velp Scientifica, Usmate, Italy) [30]. Fat content was determined according to PN-ISO 1444:2000 [31] by Soxhlet extraction method using Büchi Extraction System B-811 (Donserv, Poland).

#### 2.2.7. Cooking Yield

Steaks were grilled to a well-done degree in a convection–steam oven (Rational SCC WE61; Rational AG, Landsberg am Lech, Germany) using a steak preparation program for 9 min at 220 °C, with endpoint temperature 76 °C. Cooking yield (%) was calculated as the ratio of the sample weight after cooking to the weight before cooking.

#### 2.2.8. Texture Parameters Analysis

The texture analysis of beef steaks included shear force measurement and texture profile analysis (TPA). The tests were conducted according to the methodology described by Łozicki et al. [32] and Matyba et al. [33], using a Zwick 1120 texturometer (Zwick GmbH & Co., Ulm, Germany). Before measurement, the samples were conditioned at 20 °C to equalise their temperature. Samples for shear force measurement were cubes measuring 10 mm in height and width and 50 mm in length, cut from the centre of the steak, after removing the outer, seared surfaces. Shear force was measured using a Warner–Bratzler device with a flat knife. The maximum force required to cut through the meat sample was measured. Cuts were made across the muscle fibres while the measuring head moved at 50 mm/min. For each steak, shear force measurements were taken in six replicates, and the mean value was used as the result. Samples for TPA were cubes measuring 15 mm in height and 20 mm in length and width, cut from the centre of the steak after removing the outer, seared surfaces. The samples were compressed twice between two parallel plates, perpendicular to the muscle fibre direction, at 30% of their original height, with a 10 s relaxation period between cycles. The speed of the measurement head during the test was 50 mm/min. The springiness, cohesiveness, hardness, and chewiness of the meat samples were determined. For each steak, TPA was performed in four replicates, and the average of the individual meat texture components was taken as the result.

#### 2.2.9. Microbiological Quality

Samples of both the crust and interior parts of steaks were prepared according to PN-EN ISO 6887–2:2017 [34]. Microbiological analyses included the determination of the total plate count of aerobic mesophilic microorganisms—TPC [35], lactic acid bacteria—LAB [36], the count of Enterobacteriaceae [37], the psychrotrophic bacterial count—PBC [38] and *Pseudomonas* spp. [39], *Brochothrix thermosphacta* [40], and yeast and mould—YAM [41]. All bacterial counts were expressed as colony-forming units (log cfu) per g of product (log cfu/g).

Additionally, the presence of *Salmonella* spp. and *Listeria monocytogenes* was determined. Detection of *Salmonella* spp. in 25 g of each fraction (crust and interior) was performed using Rappaport–Vassiliadis soya broth for selective enrichment, followed by plating on Hektoen agar and Brilliant Green agar (BGA, BTL Sp. z o.o., Łódź, Poland). Presumptive colonies were subjected to preliminary characterization, including Gram staining, catalase and urease activity, and cell morphology. Isolates showing characteristics typical of Enterobacteriaceae (gram-negative, rod-shaped, catalase-positive, urease-negative) were further identified using the API 20E biochemical test system (bioMérieux, Marcy-l'Étoile, France), according to the manufacturer’s instructions. A cytochrome oxidase test was additionally performed.

The presence of *Listeria monocytogenes* in 25 g of each fraction (crust and interior) was determined using half-Fraser broth (for recovery of injured cells) followed by Fraser broth for selective enrichment, and plating on Oxford agar and ALOA medium (Agar Listeria according to Ottaviani and Agosti, BTL Sp. z o.o., Łódź, Poland). Presumptive colonies were subjected to further phenotypic characterization, including Gram reaction, catalase and urease activity, and cell morphology. Isolates showing characteristics consistent with *Listeria* spp. (gram-positive, catalase-positive, coccobacilli) were further identified using the API Listeria system (bioMérieux, Marcy-l'Étoile, France) according to the manufacturer’s instructions.

#### 2.2.10. Free Amino Acids and Biogenic Amines Profile

The materials used for free amino acid (FAA) and biogenic amine (BA) profile determination were LC-MS grade water, acetonitrile (Chemsolve), formic acid 98-100%, and ammonium formate > 99% (Chem-lab) obtained from Witko (Łódź, Poland), and trichloroacetic acid (TCA) ≥ 98% (Chempur) purchased from Pol-Aura (Zawroty, Poland). Moreover, dansyl chloride (97%) was supplied by ABCR GmbH (Karlsruhe, Germany). Sodium carbonate ≥ 99.5%, 1,7-diaminoheptane 98%, and certified analytical standards of amino acids and biogenic amines (≥97%) were obtained from Merck (Darmstadt, Germany).

The determination of free amino acids and biogenic amines in the analysed dry-aged beef steaks was performed using a modified procedure described by Świder et al. [42]. Briefly, 2 g of each sample was weighed into 50 mL centrifuge tubes. An internal standard (1,7-diaminoheptane) was added to each sample, followed by extraction with 40 mL of 5% TCA. The samples were homogenised and then centrifuged at 10,000× *g* for 10 min. The resulting extracts were filtered through filter paper. Subsequently, the extracts were derivatised: 50 µL of the extract was mixed with 2.5 mL of dansyl chloride solution (20 mM in acetonitrile) and 2.5 mL of sodium carbonate (0.05 M). The mixture was incubated in a shaking water bath at 40 °C for 1 h. The reaction was terminated by adding 10 µL of formic acid. The samples were kept in the dark for 15 min and then filtered into chromatographic vials using 0.22 µm syringe filters before LC-MS analysis.

LC-MS analysis was carried out using an Orbitrap Exploris 120 (Thermo Fisher Scientific, Waltham, MA, USA) mass spectrometer equipped with a heated electrospray ionisation (H-ESI) source. The instrument operated in positive ion mode with a spray voltage of 3.5 kV. Data acquisition was performed in full scan mode over the 200–1200 *m*/*z* range. The source parameters were set as follows: sheath gas—50 arbitrary units (Arb), auxiliary gas—13 Arb, sweep gas—1 Arb, ion transfer tube temperature—320 °C, and vaporiser temperature—300 °C.

Chromatographic separation was performed on a Raptor ARC-18 column (100 × 2.1 mm, 1.8 µm particle size; Restek, Bellefonte, PA, USA). The flow rate was 0.3 mL/min, and the injection volume was 5 µL. Mobile phase A consisted of water:acetonitrile (9:1, *v*/*v*), and mobile phase B consisted of acetonitrile:water (8:2, *v*/*v*); both phases contained 5 mM ammonium formate and 0.1% formic acid. The elution gradient was programmed as follows: 0–2 min, 0% B (isocratic); 2–22 min, linear gradient from 0% to 100% B; 22–27 min, 100% B (isocratic); 27–28 min, linear gradient from 100% to 0% B; 28–30 min, 0% B (column equilibration). Data analysis was performed using Xcalibur ver. 4.7 software (Thermo Fisher Scientific, Waltham, MA, USA). The monitored *m*/*z* values of dansyl derivatives were provided in Table S1. The results were expressed as the mean ± standard deviation (SD) of at least two measurements. The method’s linearity, expressed as the coefficient of determination (R2), was at least 0.99 for each analyte.

The values of biogenic amine indexes (BAIs) were calculated by summing the levels of cadaverine (CAD), histamine (HIS), putrescine (PUT), and tyramine (TYR) as described by Triki et al. [43].

#### 2.2.11. Sensory Evaluation

The consumer study was conducted in accordance with the principles outlined in the Declaration of Helsinki [44] and with the approval of the ethical commission (No. 15/2021). Between delivery and sensory evaluation, the set of samples was stored under refrigerated conditions at 4 °C for maximum 48 h. Prior to heat treatment, 2.0 cm-thick steaks were allowed to equilibrate to room temperature and subsequently grilled on a hot contact grill (PK2745E, 3000 W, 60 Hz, Potis GmbH, Goettingen, Germany) for 3 min at 250 °C until a medium-rare degree of doneness was achieved. After grilling, the steaks were rested for 2 min (at an average temperature of 60 °C), after which 2.0 × 2.5 cm pieces were cut perpendicular to the muscle fibres for sensory evaluation. Samples were coded with random three-digit numbers and served individually on white plates. The order of sample presentation was randomised across consumers. Crackers were provided for palate cleansing between samples.

The sensory evaluation was carried out under laboratory conditions. Participants were recruited voluntarily from individuals who declared regular steak consumption. Participants reported consuming steaks less than once per week. In total, 34 consumers (20 women and 14 men aged 22–60 years) participated in the study. Depending on session availability, the participants were divided into four groups of 6–8 consumers each. The number of participants met the formal requirements for a semi-consumer sensory assessment conducted under laboratory conditions, as described by Lawless and Heymann [45]. On each evaluation day, one or two steak samples were presented to the consumers for assessment. Sessions were typically conducted twice a week. The assessment procedures and experimental conditions were designed and carried out in accordance with the guidelines proposed by Baryłko-Pikielna and Matuszewska [46] and Meilgaard et al. [47]. The evaluated attributes included aroma before (aroma A) and after (aroma B) grilling process, tenderness, juiciness, flavour, and overall liking. All participants rated each meat sample on a 9-point hedonic category scale with end anchors ranging from 1 (extremely dislike) to 9 (extremely like), following the methodology described by Baryłko-Pikielna and Matuszewska [46].

#### 2.2.12. Identification of Fungal Species from Commercially Dry-Aged Beef Crust

Mould identification was performed only for the crust samples originating from steaks characterised by the highest overall sensory acceptance. This approach was adopted to identify fungal species potentially associated with favourable sensory attributes of dry-aged beef. Such targeted identification enables a more focused interpretation of the role of surface microbiota in desirable dry-ageing outcomes. Microbiological samples were collected by wiping a 10 × 10 cm area of the meat crust surface with a moist cotton swab. Mould cells isolated from the crust samples were cultured on potato dextrose agar (PDA; Sigma Aldrich) for 5–7 days at 25 °C. Then, colonies distinguished by morphology were re-screened to obtain a single colony. Selected colonies were further examined under the microscope to assess cellular morphology and confirm purity. DNA from pure colonies was extracted using the RNeasy PowerMicrobiome Kit (Qiagen) according to the manufacturer’s instructions. One microliter of RNase (Qiagen) was added to digest residual RNA, followed by incubation for 1 h at 37 °C. DNA concentration was measured using the Qubit dsDNA assay kit (Thermo Fisher Scientific, Waltham, MA, USA). The internal transcribed spacer (ITS1–5.8S–ITS2) region and the D1/D2 domain of the large subunit (LSU) rDNA were amplified using the primer pairs ITS5 (5′-GGAAGTAAAAGTCGTAACAAGG-3′) and ITS4 (5′-TCCTCCGCTTATTGATATGC-3′) [48], as well as NL1 (5′-GCATATCAATAAGCGGAGGAAAAG-3′) and NL4 (5′-GGTCCGTGTTTCAAGACGG-3′) [49]. The PCR products were sequenced using a 3500 genetic analyser (Applied Biosystems, Thermo Fisher Scientific, Waltham, MA, USA). The obtained ITS rDNA sequences were compared with reference sequences deposited in the NCBI GenBank database using BLASTn ver. 2.17.0. and with the UNITE fungal database. Species-level identification was assigned when sequence identity exceeded 99%, and query coverage was ≥98%. Detailed sequence similarity data, including the closest matches, accession numbers, query coverage, and identity values, are presented in Table S2. Molecular identification was also confirmed by morphological examination, including colony morphology and microscopic characteristics observed using a light microscope (Olympus CX43, Olympus Corporation, Tokyo, Japan).

#### 2.2.13. Statistical Analysis

The experimental data were expressed as mean ± standard deviation. Owing to the observational nature of the study, each commercial dry-aged beef product obtained from a different producer was treated as an observational unit. One-way analysis of variance (ANOVA) was performed to identify differences among the analysed commercial products, and significant differences among means were identified using Tukey’s HSD test (α = 0.05). To assess the effect of sampling location (crust vs. interior part), Student’s *t*-test was applied (α = 0.05).

##### Multivariate Analysis of Sensory Data

To better illustrate the obtained sensory quality results and identify clustering patterns among the tested beef steaks, cluster analysis was performed, allowing simultaneous grouping of objects and attributes. Ward’s method for agglomeration of objects and traits and the Euclidean distance measure were used.

To evaluate the relationship between aroma A and B and sensory attributes such as tenderness, juiciness, flavour, and overall palatability, canonical analysis was applied. This method enables the assessment of the relationships between two sets of variables. In this case, attributes such as aroma A and B (before and after grilling process) were included as explanatory variables, while attributes such as tenderness, juiciness, flavour, and overall palatability were treated as dependent variables.

Statistical analyses were carried out using Statistica™ software, version 13.3 PL (StatSoft Inc., Tulsa, OK, USA).

## 3. Results and Discussion

### 3.1. Physicochemical Characteristics of Dry-Aged Beef

Table 1 and Table 2 show the results of colour (L*, a* and b* parameters), water activity (a_w_), and pH of dry-aged beef steaks. Prior to colour determination, the steaks were removed from vacuum packaging and allowed to bloom for 30 min. Significant differences (*p* ≤ 0.05) were observed among steaks in all colour parameters. The analysed steaks showed variability in both lightness (L*) and redness (a*), with the greatest differentiation observed for yellowness (b*). Comparable colour parameter values for dry-aged beef were reported by Savini et al. [50]. Kim et al. [51] and Kim et al. [52] found that the a* and b* values of rump were not significantly affected by ageing time. In contrast, Álvarez et al. [53] showed that both lightness and redness are strongly affected by the duration of dry-ageing. Lancaster et al. [54] observed no significant differences in L*, a* and b* colour components among dry-aged beef samples obtained from various commercial ageing facilities in the United States, reporting L* values between 36.0 and 37.6 units, a* values between 19.2 and 21.6 units, and b* values between 13.6 and 15.7 units.

Water activity remained relatively stable across the analysed dry-aged beef steaks, with no significant differences (*p* > 0.05) observed between samples for either the crust or the interior portion (Table 2). In most cases, the water activity (a_w_) of the interior part of steaks was slightly higher than that of the crust. The dry-ageing process leads to a reduction of a_w_ at the meat surface, thereby limiting the growth of certain microorganisms. However, this effect depends on ageing conditions, such as relative humidity, temperature, ageing duration and air flow. In contrast, the water activity of the steak’s interior remains relatively high (>0.98) [4]. Savini et al. [55] have reported that the water activity of the crust obtained from beef dry-aged for 27 days ranged between 0.92 and 0.95. Cho et al. [56] observed a decrease in water activity during 60 days of the beef dry-ageing, but the differences were not significant; by the end of dry-ageing, water activity reached 0.96–0.97. Lancaster et al. [54] also found no differences in the water activity of the internal part of dry-aged beef steaks obtained from different commercial dry-ageing facilities. Although no significant differences in water activity were observed among samples, the relatively wide a_w_ range, particularly in the crust, may still be relevant from a technological and microbiological perspective, as even small differences in a_w_ can influence microbial growth dynamics during dry-ageing.

Significant differences (*p* ≤ 0.05) in pH were observed among the analysed steaks (Table 2). Although pH values of both the crust and the interior remained within a relatively narrow range in most samples, Rib-eye 2 and Rib-eye 4 were characterised by significantly higher pH values. The particularly high pH of the Rib-eye 2 and Rib-eye 4 crust may indicate spoilage-related changes. Although differences in the interior pH between samples were not significant (except Rib-eye 2), the observed wide range may still be relevant, as even moderate pH shifts can influence flavour development, microbial activity, and oxidative stability during dry-ageing and subsequent cooking. In the study by Ribeiro et al. [24], the pH of lean meat during 90 days of dry-ageing increased from 5.8 to 6.1, confirming that dry-ageing generally results in a gradual increase in pH. Meat pH influences the rate and extent of Maillard reactions during cooking, because a higher pH environment enhances browning and formation of specific flavour compounds [57]. Thus, in the context of dry-ageing beef, a moderately elevated pH could contribute to improved flavour development; however, the relationship is not strictly linear and may depend on additional factors (e.g., reducing sugar content, free amino acids, ageing time, microbial activity). Too high pH can lead to off-flavours [11]. The EFSA [21], based on a comparison of data from scientific studies and commercial practice (Section 3.2), found that most experimental studies on beef dry-ageing are performed at 0–4 °C, 75–85% relative humidity (RH), and airflow 0.2–2.5 m/s for 14–42 days. Under such conditions, the surface pH typically ranges from 5.5 to 5.9, with a surface a_w_ of 0.88 to 0.99. Similarly, Gowda et al. [4] reported a mean surface pH of 5.7 and an a_w_ range of 0.98–0.99 after evaluation of 15 Belgian dry-aged beef steaks. In the study by Kim et al. [58], the pH of beef increased from 5.7 (day 0) to 6.0 (day 60) during dry-ageing. All meat pH values were below 6.2, which is the cut-off value for the freshness of both fresh and packaged meat, as established by the Korean Ministry of Food and Drug Safety [58,59]. Overall, the pH ranges observed in our study are consistent with those reported for typical dry-aged beef. However, individual products exhibited relatively elevated crust pH values that may reflect producer-specific handling and/or microbial activity.

The values of TBARS [58,59] and water-holding capacity varied significantly (*p* ≤ 0.05) among the analysed steaks (Table 3). TBARS values showed variability between samples and, in several cases, significant differences were observed between the crust and the interior, with the crust generally exhibiting higher values. In two samples, the highest TBARS values were observed (Rib-eye 1 and Sirloin 2), indicating the most intensive lipid oxidation.

This trend results from greater exposure of beef’s outer layers to oxygen during dry-ageing. Cho et al. [56] have reported that lipid oxidation increases with ageing time; however, the TBARS values reported by these authors were <0.5 mg MDA/kg of product after 60 days of dry-ageing. Additionally, Lee et al. [60] have reported that TBARS values for dry-aged beef are increasing significantly with ageing time. The authors suggest that values > 1.00 mg MDA/kg of product are generally considered to indicate poor meat quality and, in our studies, this value was exceeded only in the crust of Rib-eye 1, Rib-eye 2 and Sirloin 2 steaks (Table 3). Elevated TBARS values may negatively affect sensory quality by contributing to rancid or stale flavour notes, especially when combined with elevated pH and high microbial activity, as observed for the Rib-eye 2 sample.

The crust of dry-aged beef steaks was characterised by low values of water-holding capacity (often 0 cm^2^/g), suggesting strong dehydration of the meat surface during dry-ageing (Table 3). The highest value of WHC (i.e., the lowest water-holding capacity) of the crust was observed for Sirloin 3, followed by the WHC of Rib-eye 4. In contrast, the WHC of the interior part of the steaks varied significantly, ranging from 0 to 19.0 cm^2^/g. In almost all cases, the interior had significantly higher WHC values than the crust, suggesting that the inner part of the meat was less dehydrated during dry-ageing. Moreover, the poorer WHC observed in leaner samples with higher water content may be related to the lower fat content, which can result in a larger area of released fluid during the filter paper press test (Table 3 and Table 4). Lee et al. [60] found that the dry-ageing method may increase the water-holding capacity of beef after 42 days of ageing.

The analysed beef steaks were characterised by significant (*p* ≤ 0.05) variability in the protein, water and fat content (Table 4).

In most samples, the crust was characterised by a protein content exceeding 20% and a water content of approximately 60%, while rib-eye steaks generally exhibited greater marbling than sirloin steaks. In most cases, no significant differences (*p* > 0.05) in protein content were found between the crust and the interior of the steaks (Table 4). There was a tendency toward higher protein content in the crust, which is associated with moisture loss during dry-ageing. This was reflected in the water content of the crust, which was lower than in the interior, although for most steaks these differences were not significant (*p* > 0.05). Fat content showed the most pronounced crust–interior differences, with the crust generally containing higher levels of fat than the interior (Table 4). In the studies of Lee et al. [60], the water, protein, and fat content of 14-day dry-aged beef varied between samples from 63.39 to 71.09%, 21.17 to 22.10% and 5.27 to 12.10%, respectively and for 21-day dry-aged beef from 58.77 to 69.14%, 19.35 to 22.01% and 6.72 to 20.01%, respectively. The authors did not find clear trends in the chemical composition of meat during dry-ageing.

In the studies by Bulgaru et al. [13], the water content in dry-aged beef decreased progressively, with a noticeable reduction of approximately 6% by day 21 and about 10% by day 35. The unique flavour characteristic of dry-aged beef results from water loss during ageing, which concentrates key meat components and enhances the development of its characteristic aged aroma [61]. According to Cho et al. [56], a decrease in water content of up to 10% after 60 days is observed during dry-ageing of beef, likely due to the protective effects of fat covering and crust formation on the meat surface. As noted by Smith et al. [62], a thicker crust can further reduce water evaporation, thereby moderating moisture loss during extended dry-ageing.

Cooking yield is an important parameter closely related to the textural characteristics and overall eating quality of meat, as moisture loss during cooking not only removes flavour compounds dissolved in water but also diminishes the moisturising effect during chewing. The cooking yield of dry-aged beef steaks in the present study ranged from 68.2 to 88.7%, with no significant differences (*p* > 0.05) observed among samples (Table 5). Nevertheless, the wide range of cooking yield values observed may still be of practical importance for both processors and food service operators, as it directly affects portion size, economic yield, and sensory juiciness perceived by consumers. Kim et al. [52] reported that cooking losses from dry-aged beef were approximately 10%, whereas Macharáčková et al. [63] and Khazzar et al. [64] found cooking losses from dry-aged striploin steaks ranging from 24.5 to 34.8% and 28.9%, respectively. In studies on dry-aged beef, WBSF and TPA have been used to monitor changes in texture during ageing, and both tests can serve as indicators of meat tenderness [13,58,65,66]. Significant differences (*p* ≤ 0.05) in WBSF values were observed, indicating variation in tenderness among beef steaks. Rib-eye 11 and Rib-eye 8 were characterised by the highest tenderness, whereas some samples remained relatively tough. Despite the dry-ageing process, some samples remained relatively tough, suggesting that the tenderization effect was not achieved. Such large differences between samples may result from complex interactions among muscle fibre composition, connective tissue content, and individual ageing dynamics. Moreover, Tejrung et al. [12] described the so-called “dry-aged beef paradox”, emphasising that although dry-ageing develops a distinctive and desirable flavour profile, it does not always guarantee an improvement in meat texture. Hulánková et al. [67] reported that beef ageing generally improves tenderness. The authors observed a decrease in shear force values in the loin muscle during dry-ageing. Similarly, Kim et al. [58] found that beef aged for 15 days was more tender than beef analysed on day 0, confirming that the degree of tenderization strongly depends on the duration of ageing. Moreover, even when biostarters were used for dry-ageing beef, after 28 days the beef was very tender and juicy, though the biostarter-inoculated samples did not show significantly higher tenderness (lower shear force) or juiciness than the control samples. The enhancement of these sensory attributes primarily results from the natural progression of the dry-ageing process rather than from the use of biostarters [28].

Cohesiveness reflects the strengths and weaknesses of the binding functions of molecules or structural elements in the samples, and springiness is a mechanical textural attribute related to the rapidity and degree of recovery from a deforming force [68]. There were no significant differences (*p* > 0.05) in cohesiveness and springiness between beef steaks (Table 5). This was consistent with the results of Kim et al. [58], who also found no differences in these texture parameters among samples, regardless of the ageing method or ageing time. Although significance was not reached, variability in cohesiveness and springiness may still contribute to subtle differences in mouthfeel and overall eating quality, which can be relevant in premium products such as dry-aged beef. Hardness of beef steaks differed significantly between samples (*p* ≤ 0.05). The tendency observed in Rib-eye 11 was typical of aged beef: tender but with a compact, firm surface due to moisture loss during ageing.

Chewiness refers to the energy required to chew solid samples and comprehensively reflects the continuous resistance of the samples to chewing [68]. As with hardness, significant differences (*p* ≤ 0.05) in chewiness were observed among samples. High chewiness values were generally associated with greater sample hardness (Table 5).

### 3.2. Microbiological Quality of Dry-Aged Beef

Table 6 presents the microbiological quality of the crust and interior of commercially available dry-aged beef steaks from Poland, including total plate count (TPC), psychrotrophic bacteria count (PBC), and lactic acid bacteria (LAB). Relatively high and variable microbial loads characterised the microbiological quality of the analysed steaks. Significant differences in TPC were observed among samples, reflecting the heterogeneous conditions of commercial dry-ageing practices, including differences in ageing time and conditions such as temperature, airflow, and relative humidity, as well as hygiene management. In most cases, the crust exhibited significantly higher (*p* ≤ 0.05) TPC values than the interior, which is characteristic of dry-ageing processes, where prolonged exposure to oxygen and controlled dehydration promote the development of surface microbiota. Despite this, TPC values in the interior portions of the steaks, representing the edible part of the product, remained within the range commonly reported for beef and suggest satisfactory microbiological quality [51,69,70]. There are currently no specific microbiological criteria established for TPC in dry-aged beef.

The dominance of PBC is consistent with the low-temperature conditions during dry-ageing, which favour the growth of cold-tolerant microorganisms. Significant differences in PBC values were observed among samples (Table 6). For the majority of steaks, PBC values were significantly higher (*p* ≤ 0.05) on the crust than in the interior. Psychrotrophic bacteria are known to contribute to proteolytic and lipolytic processes, and may therefore play a role in the development of the characteristic flavour of dry-aged beef [51].

LAB counts differed significantly among the analysed samples (Table 6). For most samples, LAB values in the interior were significantly lower than those in the crust, ranging from 2.30 log cfu/g to 4.70 log cfu/g. The presence of LAB in dry-aged beef is considered technologically relevant due to their antagonistic effects against spoilage and pathogenic microorganisms, and their potential contribution to microbial stability during ageing [8].

Table 7 presents Enterobacteriaceae, yeasts and moulds (YAMs), *Brochothrix thermosphacta*, and *Pseudomonas* spp. counts of the crust and interior of commercial dry-aged beef steaks from Poland. Enterobacteriaceae counts were generally low in both the crust and the interior (from <1.00 log cfu/g to 4.27 log cfu/g) indicating satisfactory hygienic quality of the raw material and processing conditions. Significantly higher (*p* ≤ 0.05) Enterobacteriaceae counts were observed on the crust than in the interior (Table 7). Enterobacteriaceae are reported at very low levels in dry-aged beef, and several studies have described their absence or levels below the detection limit in surface samples. Hulánková et al. [67] and Ryu et al. [16] have reported that Enterobacteriaceae, coliforms, and *Escherichia coli* were not detected on the surface of dry-aged beef loins under the applied ageing conditions. Long-term ageing studies have reported only moderate changes in Enterobacteriaceae levels over extended ageing. Smaldone et al. [71] observed a gradual decrease in Enterobacteriaceae counts from approximately 2.1 log cfu/g to 2.6 log cfu/g during 277 days of ageing. Conversely, Khazzar et al. [64] reported a moderate increase from about 1 log cfu/g to 1.7 log cfu/g after 230 days of ageing. Similarly, Di Paolo et al. [66] observed an increase in Enterobacteriaceae from approximately 1.8 log cfu/g to 1.9 log cfu/g between day 2 and day 60 of beef dry-ageing.

Yeasts and moulds (YAMs) are commonly observed on the crust of dry-aged beef steaks. They may contribute to the development of characteristic sensory attributes of such meat through their enzymatic activity [72]. In the present study, YAM counts on the crust were significantly differentiated (*p* ≤ 0.05) among samples, reflecting variations in ageing and storage conditions between producers.

The presence of yeasts and moulds on the crust is typical of dry-aged beef [72]. Dry-ageing can encourage the growth of moulds tolerant to reduced water activity [16]. Investigations of commercial dry-aged loins have detected moulds on the dried surface of about half of samples aged for 19 days or longer, and the numbers were generally low, while yeasts were often present in the majority of samples, further confirming frequent fungal colonization of the crust [4]. Smaldone et al. [71] reported the presence of yeasts and moulds on dry-aged beef surfaces at the level of 2 log cfu/g. Similarly, Di Paolo et al. [66] observed that total yeast and mould counts remained relatively stable over 60 days of ageing, with yeast numbers increasing only slightly from approximately 3.6 log cfu/g to about 4.8 log cfu/g.

*Brochothrix thermosphacta* was detected at relatively high levels on the crust of dry-aged beef steaks, with high variability among samples (Table 7). In the majority of samples, *B. thermosphacta* counts were significantly (*p* ≤ 0.05) higher on the crust than in the interior. The interior parts of the steaks showed lower and less variable *B. thermosphacta* counts, with the highest levels detected in the Rib-eye 4 steak. Although *B. thermosphacta* is recognised as a major spoilage organism in chilled meats, producing metabolites associated with quality deterioration and off-odours during refrigerated storage [73], its predominance on the crust suggests that its activity during dry-ageing is primarily confined to the surface, thereby limiting its impact on the microbiological quality of the edible portion of the dry-aged meat. This distribution pattern is consistent with observations by Liu et al. [74], who reported that the absence or low abundance of *B. thermosphacta* in the interior may be due to limited growth under oxygen-deficient conditions and a restricted ability to penetrate deeper muscle tissues. In contrast, the relatively high counts of *Brochothrix* observed in the interior of selected samples may be attributed to secondary contamination, for example, during trimming, when hygienic conditions are not fully controlled. The EFSA [21] have emphasised that cutting and trimming must follow strict sanitary procedures to prevent microbial contamination.

*Pseudomonas* spp. are among the dominant aerobic microorganisms associated with fresh and aged beef surfaces [67]. *Pseudomonas* spp. counts on the crust of dry-aged beef were significantly differentiated. Significantly lower (*p* ≤ 0.05) counts were observed in the interior parts of steaks (Table 7). According to Yang et al. [75], *Pseudomonas* spp. counts in the range of 7–8 log cfu/g are typically associated with undesirable alterations in meat quality, including colour changes, slime formation and the development of unpleasant odours. Despite their high surface abundance, the limited presence of *Pseudomonas* spp. in the interior suggests a reduced risk of spoilage-related quality deterioration in the edible portion following crust removal.

Higher microbiological loads observed on the crust in our studies are consistent with studies reporting intensive microbial colonisation of exposed meat surfaces during dry-ageing, where oxygen availability and moderate dehydration favour the growth of aerobic microorganisms. In contrast, the inner muscle tissue, which constitutes the edible portion of the meat, remains relatively protected due to dehydration of the outer layers, its reduced water activity, and limited oxygen diffusion, which collectively inhibit microbial penetration into deeper muscle layers [24,51,69,70]. As a result, the interior of dry-aged beef exhibits relatively low and stable microbial counts throughout the ageing period, confirming the effectiveness of the crust as a physical and microbiological barrier [69,70]. The variability observed among commercial samples probably reflects the non-standardised conditions of dry-ageing; nevertheless, the overall microbiological quality of the edible portion remains acceptable when appropriate trimming and hygienic handling practices are applied.

API Listeria and API 20E analyses did not confirm the presence of *Listeria monocytogenes* or *Salmonella* spp. in the analysed dry-aged beef samples. The following microorganisms were identified in the analysed dry-aged beef samples: *Hafnia alvei* (99.9%) in Rib-eye 2 crust, Rib-eye 8 crust, and Sirloin 1 interior. An inconclusive API 20E profile in Rib-eye 6 crust showing probabilities for *Shigella* spp. (36.1%), *Acinetobacter* spp. (28.1%), and *Tatumella ptyseos* (21.8%); *Hafnia alvei* (91.6%) in Rib-eye 7 interior; and *Listonella damsela* (98.9%) in Sirloin 3 crust. The predominance of environmental or opportunistic Enterobacteriaceae, particularly *Hafnia alvei*, is consistent with previous observations that the surface microbiota of dry-aged beef is shaped by aerobic conditions, prolonged storage, and reduced water activity. At the same time, the absence of confirmed *Listeria monocytogenes* and the lack of clear confirmation of *Salmonella* spp. indicate that the applied dry-ageing conditions did not favour the proliferation of these major foodborne pathogens. Overall, these findings suggest that, although surface-associated microbial activity was evident, the microbiological status of the analysed dry-aged beef remained acceptable, particularly considering that the crust is removed prior to consumption.

### 3.3. Free Amino Acid and Biogenic Amines Profile of Dry-Aged Beef

In this discussion, particular attention was given to changes in the profile of free amino acids that serve as direct precursors of biogenic amine formation (Table 8). The concentrations of the remaining determined free amino acids are reported in Supplementary Tables S3–S5. The contents of biogenic amines in the crust and interior parts of dry-aged steaks, including histamine (HIS), cadaverine (CAD), putrescine (PUT), and tyramine (TYM), along with the calculated biogenic amines index (BAI), are presented in Table 9, while the remaining biogenic amines are shown in Supplementary Table S6. The contents of amino acids and biogenic amines showed significant variability among steaks as well as between the crust and the interior of dry-aged beef. These differences likely reflect the combined effects of muscle proteolysis, surface dehydration, and spatially differentiated microbial activity occurring during the dry-ageing process [15,76].

Histidine content varied significantly among steaks, while the significant crust–interior differences were observed only in selected samples, such as Rib-eye 11 (Table 8). In the available literature, histidine, the precursor of histamine, has been reported to increase in dry-aged beef during ageing, reflecting ongoing proteolysis and concentration effects from moisture loss, reaching 132 mg/kg after 28 days of ageing [77].

Histamine (HIS) is produced by bacterial decarboxylation of histidine via L-histidine decarboxylase. In the present study, significant (*p* ≤ 0.05) differences in HIS content were observed between steaks, both in the crust and in the interior (Table 9). Significant crust–interior differences (*p* ≤ 0.05) were detected only in a limited number of samples; however, in all samples, HIS content was higher in the crust. This tendency may be associated with the higher abundance of surface-associated microbiota, including Enterobacteriaceae, a bacterial group linked to the production of several biogenic amines, such as HIS, CAD, and TYM [78]. According to Balamatsia et al. [79], HIS formation is typically associated with meat spoilage, particularly when Enterobacteriaceae populations reach approximately 7 log cfu/g, a level not observed in the present study. Similarly, Ribeiro et al. [80] reported low HIS levels (<5 mg/kg) in 60-day dry-aged beef. Despite the availability of histidine (Table 8), HIS levels remained low across most samples (Table 9), suggesting limited activity of histidine-decarboxylating microorganisms, which is consistent with the relatively moderate levels of Enterobacteriaceae observed (Table 7). Therefore, the tendency toward higher histamine levels in the crust of steaks appears to be primarily associated with localised microbial activity on the surface rather than with differences in histidine availability alone.

Lysine levels differed significantly among steaks and between the crust and interior (Table 8), although the direction of crust–interior differences was not consistent across samples. In other studies [81,82] lysine content in dry-aged beef was also shown to increase during ageing. Lysine is a direct precursor of cadaverine (CAD), a biogenic amine formed by microbial decarboxylation [81]. Cadaverine commonly accumulates in meat during storage and ageing and is often associated with increased bacterial activity [11]. Enterobacteriaceae and certain spoilage-associated bacteria, such as *Pseudomonas* spp. are recognised for their ability to decarboxylate lysine to CAD [83].

In the present study, cadaverine levels showed significant variability among steaks, and for several steaks CAD levels were significantly higher (*p* ≤ 0.05) in the crust than in the interior. This can be attributed to higher microbial loads (Enterobacteriaceae and *Pseudomonas* spp.) on the crust (Table 7). In the studies by Ribeiro et al. [80], CAD levels at 35 and 60 days of ageing were 5.4 and 31.1 mg/kg, respectively, in the interior part of the meat. Despite relatively high lysine availability in several samples (Table 8), CAD levels varied and were not strictly proportional to lysine content, suggesting that precursor availability alone was not the limiting factor for cadaverine formation. The higher CAD levels observed predominantly on the crust are more likely explained by enhanced microbial activity in this fraction, as supported by higher Enterobacteriaceae counts on the crust than in the interior (Table 7). This is consistent with the notion that CAD accumulation in dry-aged beef is primarily driven by the activity and distribution of decarboxylase-positive microorganisms rather than solely by lysine availability within the muscle tissue [77].

The content of ornithine, a non-proteinogenic amino acid formed primarily through arginine metabolism and one of the main precursors of putrescine (PUT), significantly differed between steaks (Table 8). Significant crust–interior differences were observed in several samples, with higher ornithine concentrations detected on the crust. This is consistent with intensified microbial metabolism at the exposed surface of beef during dry-ageing (Table 7).

Putrescine is one of the most frequently reported biogenic amines in meat and is commonly associated with microbial decarboxylation of ornithine and arginine during storage and ageing [79]. In the present study, PUT concentrations varied significantly (*p* ≤ 0.05) among steaks and between the crust and interior (Table 9). In several samples, significantly higher (*p* ≤ 0.05) PUT levels were observed in the crust when compared with the interior, whereas the interior was characterised by lower and less variable levels, indicating limited formation in deeper muscle tissues. The higher PUT levels on the crust may be associated with the higher microbial loads (LAB, Enterobacteriaceae, *Pseudomonas* spp.) observed on the exposed surface of dry-aged beef (Table 7). This distribution corresponded with the higher ornithine concentrations found in the same fraction, indicating that surface conditions may favour this metabolic pathway (Table 9).

Although PUT and CAD are generally not considered highly toxic at concentrations typically detected in meat, they are widely used as indicators of spoilage because they contribute to unpleasant putrefactive odours and are closely associated with microbial activity [84]. However, no specific concentration thresholds for putrescine and cadaverine associated with toxicity or sensory rejection in meat products have been established in the literature.

Tyrosine levels varied significantly (*p* ≤ 0.05) among the analysed steaks and between the crust and the interior (Table 8). In the majority of steaks, tyrosine levels were higher on the crust than in the interior, with significant crust–interior differences observed in selected samples. This distribution pattern indicates more intensive proteolytic release of aromatic amino acids in the outer layers of dry-aged beef. Previous studies on dry-aged beef have similarly reported higher concentrations of free aromatic amino acids, including tyrosine, on exposed meat surfaces due to intensified proteolysis and microbial metabolism during beef ageing [76,85].

Tyrosine is the direct biochemical precursor of tyramine (TYM), produced by microbial decarboxylation and primarily mediated by LAB and certain spoilage microorganisms [79,84]. In our study, TYM was the predominant monoamine detected in the analysed steaks and also showed significant variability among samples (*p* ≤ 0.05; Table 9). TYM concentrations were higher on the crust than in the interior, with significant (*p* ≤ 0.05) crust–interior differences observed in selected samples, and this tendency corresponded with the higher tyrosine content on the crust (Table 8). The tendency toward higher TYM levels on the crust is consistent with increased microbial activity (LAB) in the crust of the analysed beef (Table 7).

Large differences in PUT, CAD, and TYM concentrations observed among individual steaks may result from the combined influence of several technological, microbiological, and raw-material-related factors inherent to commercial dry-ageing practices. Dry-ageing is typically performed under non-standardised conditions, and differences in ageing time, temperature, relative humidity, and airflow between producers or even within batches can substantially affect microbial growth and metabolic activity on the meat surface. Such variability may lead to differences in the extent of amino acid decarboxylation and, consequently, in the accumulation of biogenic amines [12,60,67].

The sum of HIS, CAD, PUT, and TYM can be used to assess meat freshness, and it is commonly referred to as BAI. Based on the BAI values, meat quality can be classified from good to spoiled. Although no regulatory limits have been established for BAI in beef, proposed reference values in the literature vary widely by technological context. Lower threshold values (approximately 50 mg/kg) have been suggested for fresh meat as indicators of early spoilage and loss of freshness [43]. However, such criteria do not apply to dry-aged beef, where biogenic amine formation is an inherent consequence of prolonged ageing and surface microbial activity, and BAI can reach several hundred mg/kg. In this context, the high BAI values observed in the crust reflect intensified surface-associated biochemical activity during dry-ageing and do not indicate a safety concern, as this part is not intended for consumption. The lower BAI values recorded in the interior confirm that the edible portion of dry-aged beef remained biochemically stable.

The predominance of biogenic amines on the crust (Table 9 and Table S6) is consistent with microbial decarboxylation activity associated with surface microbiota, particularly aerobic spoilage bacteria. While general microbiological profiling studies confirm higher counts on exposed surfaces during dry-ageing [24], literature on biogenic amines highlights that compounds such as PUT, CAD, and TYM accumulate primarily where microbial enzyme activity is highest [86].

### 3.4. Sensory Quality of Dry-Aged Beef

**Figure 1:**
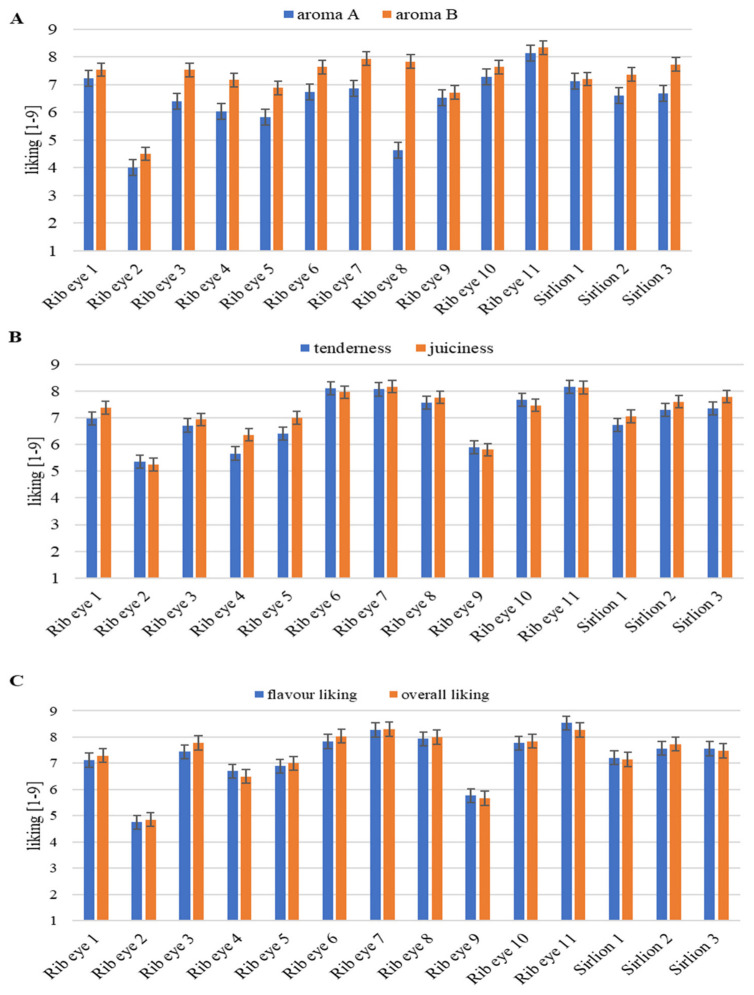
Mean values with standard deviation of the sensory liking of studied samples of beef steaks ((**A**)—odour notes, (**B**)—tenderness and juiciness notes, (**C**)—flavour liking and overall liking notes (hedonic scale 1–9; *n* = 34)).

The results of the consumer sensory evaluation of commercially available dry-aged beef steaks in Poland are presented in Figure 1. Colour and aroma are a key sensory attributes that shapes consumers’ first impressions of meat quality and strongly influences purchase decisions and acceptance. Unpleasant or atypical aroma characteristics are among the main factors leading to consumer rejection. In the present study, odour scores differed between steaks, both before (aroma A) and after grilling (aroma B). The highest scores for both aroma attributes were given to Rib-eye 11 steak, indicating high odour desirability regardless of thermal processing (Figure 1A). In contrast, Rib-eye 2 steaks received the lowest scores for aroma A and aroma B, suggesting poor aroma acceptance that persisted even after grilling. Several samples (e.g., Rib-eye 8) showed a clear improvement in aroma desirability after grilling, indicating that thermal processing enhanced their sensory aroma profile. Consumers described samples with lower raw-odour desirability as having bloody or pungent notes, characteristics more typical of wet-aged beef than dry-aged beef, despite the producers’ declarations.

Tenderness and juiciness were indicated as the most important attributes determining the overall desirability and consumer acceptance. As shown in Figure 1B, the highest scores for both tenderness and juiciness were given for samples Rib-eye 6, Rib-eye 7, Rib-eye 8, Rib-eye 10, and Rib-eye 11, whereas lower scores for these attributes characterized Rib-eye 2 and Rib-eye 9.

Flavour liking and overall liking scores (Figure 1C) followed a similar pattern. The highest ratings were observed for Rib-eye 6, Rib-eye 7, Rib-eye 8, Rib-eye 10, and Rib-eye 11, indicating that these steaks exhibited the most desirable overall sensory profiles and the greatest potential for consumer acceptance. In contrast, Rib-eye 2 and Rib-eye 9 consistently received lower scores, reflecting inferior palatability. These findings are consistent with studies indicating that the primary objective of dry-ageing is to enhance tenderness and intensify flavour.

As Marie-Pierre et al. [87] found, dry-ageing leads to reduced shear force, increased juiciness, higher dry matter content, and concentration of flavour compounds, resulting in characteristic sensory notes described as beefy, nutty, and buttery. That is why the lovers of mature dry beef are willing to pay more for it. Similarly, Lee et al. [72] have reported that ageing is primarily applied to improve tenderness, juiciness, and overall palatability, effects that were clearly achieved for several of the steaks evaluated in the present study.

**Figure 2:**
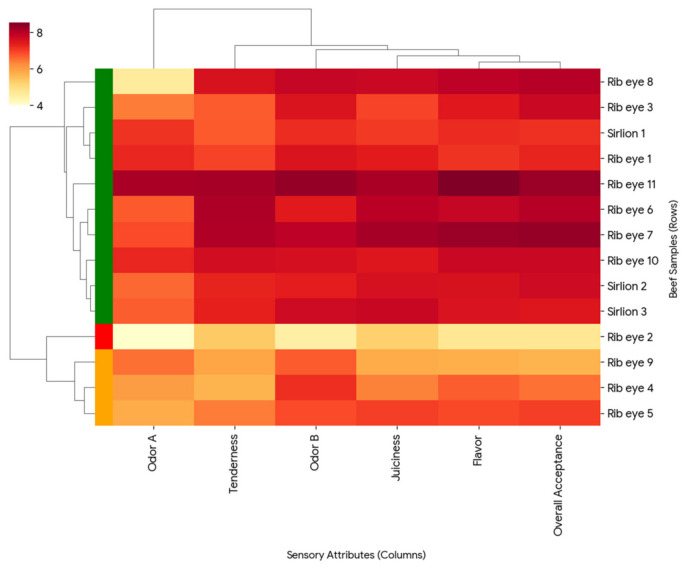
Results of a two-way hierarchical cluster analysis (clustermap) of sensory profiling data for dry-aged beef. Column dendrogram groups sensory attributes based on correlation (odour A/B, tenderness, juiciness, flavour, and overall acceptance). The row dendrogram classifies beef cuts (rib-eye and sirloin) by quality profile. Side colour bar specifies the identified clusters: green indicates premium quality, orange represents medium quality, and red highlights the highly deficient outlier (Rib-eye 2). The colour intensity ranges from light yellow (low sensory liking) to dark red (high sensory liking).

To further explore relationships among steaks and sensory traits, two-way hierarchical cluster analysis was performed using Ward’s variance-minimisation method and Euclidean distance, allowing simultaneous grouping of samples and sensory traits (Figure 2).

The samples clustering successfully partitioned the beef into three distinct quality clusters. Premium cluster (green) comprised the majority of the samples (e.g., Rib-eye 11, Rib-eye 10, Rib-eye 8, Rib-eye 7, Rib-eye 6, Rib-eye 3, Rib-eye 1, as well as Sirloin 1, Sirloin 2, Sirloin 3), which were characterised by high sensory scores across all evaluated attributes. The medium cluster (orange), including Rib-eye 4, Rib-eye 5, and Rib-eye 9, was associated with intermediate ratings, particularly for juiciness and flavour. Rib-eye 2 beef formed a separate single-element cluster, indicating an atypical sensory profile characterised by lower scores for flavour, juiciness, and tenderness, which resulted in reduced overall consumer acceptance.

To evaluate whether aroma attributes could predict texture-related and hedonic properties, canonical correlation analysis was performed. Aroma A and aroma B were included as explanatory variables, whereas tenderness, juiciness, flavour liking, and overall liking were the dependent variables. A highly significant canonical correlation coefficient (CR = 0.95; *p* < 0.01) was obtained, accounting for 90.35% of the shared variance between the two sets of variables. Partial correlation analysis revealed a particularly strong association between aroma after grilling (aroma B) and flavour liking (r = 0.93; *p* < 0.01). These results indicate that aroma development during grilling plays a central role in shaping consumers’ perception of texture and palatability. Consequently, the controlling factors influencing aroma formation during dry-ageing are critical for achieving high sensory quality. This conclusion is consistent with the synthesis by Ribeiro et al. [7], who described the characteristic bouquet of dry-aged beef, often perceived as beefy, nutty, buttery, and earthy, as resulting from complex biochemical and microbial processes. Properly controlled ageing conditions, therefore, allow the development of distinctive aromas closely linked to superior texture and eating quality.

The sensory evaluation revealed clear differences in the acceptance of the analysed dry-aged beef steaks, which could be linked to their physicochemical and microbiological characteristics. Samples with the highest sensory scores were generally characterised by a favourable balance between ageing processes and controlled microbial activity. In contrast, samples with excessive microbial loads tended to show reduced sensory acceptance. In particular, the Rib-eye 11 steak was identified as the sample with the most favourable overall quality profile. This steak combined high consumer scores for flavour, tenderness, and overall liking with relatively low counts of Enterobacteriaceae, *Pseudomonas* spp., and *Brochothrix thermosphacta*. Moreover, Rib-eye 11 showed a moderate pH and low TBARS levels, indicating limited lipid oxidation. The simultaneous presence of a high content of free amino acids allows the development of desirable sensory attributes without the formation of off-flavours. In contrast, Rib-eye 2 steak was classified as the sample with the least favourable overall quality. Despite some physicochemical parameters remaining within acceptable ranges, this sample was characterised by high microbial loads, particularly total viable counts and elevated levels of *Pseudomonas* spp. and *Brochothrix thermosphacta*. Additionally, the high pH may have further promoted microbial and enzymatic activity associated with spoilage processes. These microorganisms are known to contribute to the formation of undesirable sensory notes, such as sour, dairy-like, or putrid odours, which are likely to negatively influence consumer perception and overall sensory acceptance. These samples were also characterised by the highest fat content and one of the highest TBARS index. Although Rib-eye 2 exhibited the highest absolute concentrations of free amino acids associated with umami and flavour enhancement, including glutamic acid and aspartic acid (Tables S3 and S4), this sample was simultaneously characterised by markedly elevated levels of amino acids indicative of degradation processes, such as histidine, lysine, and ornithine (Table 8). The predominance of these degradation-related amino acids suggests intensified microbial and enzymatic activity, which may have negatively affected sensory perception and masked the positive contribution of umami-related compounds. In contrast, Rib-eye 11 showed a more favourable balance between free amino acids associated with umami (e.g., glutamic and aspartic acids; Tables S3 and S4) and those linked to degradation processes (Table 8). Despite lower absolute concentrations of umami-related amino acids compared with Rib-eye 2, this balanced free amino acid profile is consistent with the superior sensory acceptance observed for Rib-eye 11.

### 3.5. Mould (Filamentous Fungi) Identification in Dry-Aged Beef Crust

**Figure 3:**
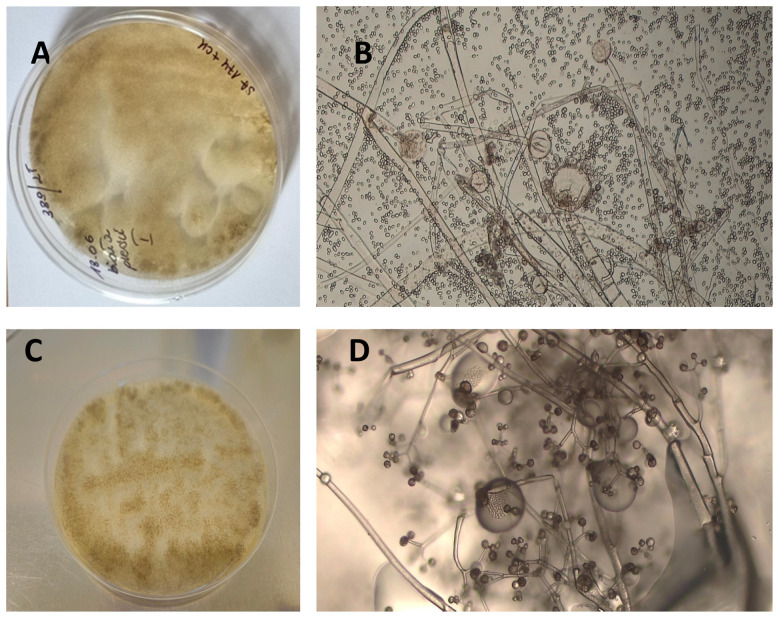
Morphological characteristics of *Mucor flavus* and *Thamnidium elegans* isolates. Colony appearance (**A**) and microscope image (**B**) of *Mucor flavus*; colony appearance (**C**) and microscope image (**D**) of *Thamnidium elegans*.

The surface of dry-aged meat is known to harbour a complex fungal community, particularly xerotolerant and xerophilic moulds adapted to low water activity and elevated salt concentrations [88]. In the present study, ITS rDNA sequencing combined with morphological characterisation enabled reliable identification of seven filamentous fungal species: *Mucor flavus* (Rib-eye 3), *Phoma herbarum* (Rib-eye 1), *Thamnidium elegans* (Rib-eye 11), *Aureobasidium melanogenum* (Rib-eye 1), *Irpex laceratus* (Rib-eye 11), *Penicillium palitans* (Rib-eye 8, Rib-eye 10), and *Penicillium solitum* (Rib-eye 8) (Figure S1 and Figure 3).

Phylogenetic analysis indicated that the isolates belonged to taxonomically diverse genera within three major fungal phyla: Mucoromycota, Ascomycota, and Basidiomycota. *Mucor flavus* and *Thamnidium elegans* were classified within the phylum Mucoromycota (family Mucoraceae), taxa frequently associated with meat and meat-processing environments, particularly under refrigerated conditions [23]. Members of Mucoraceae are naturally and commonly present on beef surfaces and have been reported to influence dry-ageing processes [23]. Species assigned to the phylum Ascomycota included *Phoma herbarum*, *Aureobasidium melanogenum*, *Penicillium palitans*, and *Penicillium solitum* [89].

From a technological perspective, the most interesting fungal species isolated from dry-aged beef crust in our study were *Mucor flavus* and *Thamnidium elegans* (Figure 3). Moreover, *Thamnidium elegans* was isolated from steaks that received the highest scores in sensory evaluation (Figure 1 and Figure 2).

The presence of these species is particularly important for the quality of dry-aged beef [90]. Although members of the genus *Mucor* are generally regarded as opportunistic fungi, selected species have been investigated for technological applications in food production. In particular, *Mucor flavus* has been successfully used as a biostarter during the dry-ageing of beef [10,15,90,91,92,93,94,95]. Przybylski et al. [91] have showed that inoculation with the *Mucor flavus* KKP 2092p significantly improved the sensory quality of dry-aged beef, allowing high sensory quality to be achieved after just 21 days. Compared with the control, the inoculated meat exhibited a distinct profile of volatile organic compounds. Moreover, Przybylski et al. [20] reported that the use of *Mucor flavus* KKP 2092p during the 21-day dry-ageing process resulted in lower post-grilling shear force values. Hanagasaki and Asato [93] have reported that the use of *Mucor flavus* as a starter culture reduced weight loss during the dry-ageing process. Meat surfaces fully colonized by the mould exhibited lower ageing losses, with weight reduction remaining below 30% between the third and fourth weeks of ageing, compared with non-inoculated controls. Furthermore, Mikami et al. [10] investigated the influence of fungal communities comprising *Mucor flavus* and *Helicostylum pulchrum* on the sensory properties of dry-aged beef. Their findings indicated that the presence of these moulds contributed to the development of desirable aroma attributes, including mushroom-like and nutty notes, resulting in an overall improvement in flavour quality. Collectively, these findings indicate that selected *Mucor* strains may positively influence the technological and sensory quality of dry-aged beef and therefore represent promising fungal biostarters. Nevertheless, its safety should be evaluated on a strain-specific basis, particularly when intended for food applications.

*Thamnidium* spp. are frequently dominant moulds during dry-ageing [7]. *Thamnidium elegans* is a cold-tolerant mould species that naturally develops on the surface of beef during dry-ageing, and its growth can begin as early as 3 weeks after the ageing process starts [95]. This mould is typically observed as a pale grey, filamentous growth that forms characteristic whiskers on the fat-rich areas of the meat surface [3]. *Thamnidium elegans* is of particular interest in food production because of their proteolytic and lipolytic properties. Itsability to secrete collagenolytic enzymes promotes the degradation of perimysial connective tissue, thereby improving meat tenderness beyond the effects of endogenous proteases, including calpains [10,93,94].

On the other hand, representatives of *Penicillium* are commonly detected on dry-aged beef crusts, alongside other fungi such as *Penicillium camemberti*, *Debaryomyces hansenii*, and *Pichia anomala*, which have previously been reported in Asian dry-aged beef production systems [9,23,60]. In the present study, two *Penicillium* species, *P. palitans* and *P. solitum*, were identified. Both species have previously been reported as members of the mycobiota associated with traditional dry-cured meat products, including fermented sausages and dry-cured beef [96,97,98]. However, some *Penicillium* species are known to produce mycotoxins, including ochratoxin A (OTA), cyclopiazonic acid (CPA), patulin (PAT), and mycophenolic acid (MPA) [21,96,98]. For example, *P. polonicum* has been reported to produce toxic OTA and citrinin (CIT) under specific environmental and substrate conditions and has been isolated from meat products while potentially posing a risk to consumers [99,100,101,102]. Similarly, *P. verrucosum* and *P. nordicum* are recognized producers of OTA in dry-cured meat products [103]. Ribeiro et al. [96] have reported that eight *Penicillium* species isolated from *Cecina de León*, a traditional Spanish dry-cured beef product, were capable of producing at least one mycotoxin. Among these, *P. commune* and *P. palitans* were identified as potential producers of CPA, whereas *P. solitum*, the second most frequently isolated species, was reported to produce MPA. In addition, *P. griseofulvum* was shown to produce several mycotoxins, including CPA, PAT, and griseofulvin (GRI).

In contrast, *Irpex laceratus*, isolated from the crust surface in the present study, most likely represents environmental contamination [104]. This basidiomycetous fungus is typically associated with plant-derived substrates but may colonize meat surfaces via air exposure during ageing [105]. Although *Irpex laceratus* has not been associated with dry-aged meat production, recent studies have revealed its capacity to synthesize a variety of volatile and secondary metabolites exhibiting potential antimicrobial and other biologically active properties [106]. Nevertheless, no data are currently available regarding its influence on the quality, safety, or sensory attributes of dry-aged meat.

In summary, the fungal community identified in the present study was largely consistent with the mycobiota previously reported in dry-aged beef and other dry-cured meat products. The detected species likely originated from both natural environmental colonization during ageing and fungal populations adapted to meat-processing environments. While some fungi, such as *Mucor flavus* and *Thamnidium elegans*, have been associated with desirable technological effects, including improved flavour development and meat tenderization, the presence of potentially mycotoxigenic species highlights the importance of continuous microbiological surveillance. As certain *Penicillium* species identified in meat ecosystems have been reported to produce mycotoxins under favourable conditions, monitoring fungal diversity and controlling environmental parameters during processing remain essential.

## 4. Conclusions

The present study demonstrates variability among dry-aged beef from different producers. This variability concerned physicochemical traits, texture, microbiological status, free amino acid profiles, biogenic amine accumulation, sensory quality, and the composition of crust-associated filamentous fungi. Moreover, differences were observed between the crust and the interior of the analysed steaks. The crust was characterised by lower water activity, higher lipid oxidation, higher microbial loads, and generally higher concentrations of biogenic amines. In contrast, the interior of the meat remained more stable in terms of physicochemical parameters and microbiological status and was characterised by lower concentrations of biogenic amines. The results also showed that the accumulation of biogenic amines was more strongly associated with the microbial activity on the crust than with the availability of free amino acid precursors alone. Despite the presence of diverse surface microbiota, no confirmed occurrence of *Salmonella* spp. or *Listeria monocytogenes* was detected in the analysed samples, indicating that properly conducted commercial dry-ageing does not inherently increase the risk associated with these major foodborne pathogens. At the same time, the crust should be considered a critical zone requiring strict hygienic control and appropriate trimming before consumption. The sensory evaluation indicated that selected steaks achieved high consumer acceptance, particularly in terms of aroma after grilling, tenderness, juiciness, flavour liking, and overall liking. The identification of fungal species such as *Mucor flavus* and *Thamnidium elegans* in crust samples from highly rated steaks suggests that some members of the dry-aged beef mycobiota may play a beneficial technological role and contribute to desirable quality attributes.

A limitation of the present study is the lack of detailed information on animal characteristics and detailed dry ageing conditions applied by individual producers. Therefore, future studies conducted under controlled ageing conditions are needed to determine the contribution of specific factors on dry aged beef quality and safety.

The findings confirm that dry-aged beef available on the Polish market can represent a high-quality and microbiologically safe product; however, its final quality depends strongly on producer-specific practices. These results highlight the need for greater standardization of dry-ageing conditions and confirm the importance of monitoring the crust separately from the edible interior when assessing the quality and safety of dry-aged beef.

## Figures and Tables

**Table 1:** Colour parameters of the commercial dry-aged beef steaks from Poland.

Steak Type	Colour Parameters		
	L*	a*	b*
Rib-eye 1	37.9 ^ab^ ± 1.5	21.1 ^ab^ ± 1.8	9.2 ^cde^ ± 0.7
Rib-eye 2	45.0 ^b^ ± 2.2	14.9 ^a^ ± 0.5	8.2 ^cd^ ± 0.8
Rib-eye 3	33.8 ^a^ ± 0.9	22.8 ^b^ ± 2.3	10.3 ^cde^ ± 0.4
Rib-eye 4	32.4 ^a^ ± 3.2	16.2 ^a^ ± 1.1	5.1 ^a^ ± 0.5
Rib-eye 5	38.5 ^ab^ ± 2.7	23.0 ^b^ ± 0.4	11.2 ^e^ ± 1.1
Rib-eye 6	39.2 ^ab^ ± 3.5	17.6 ^ab^ ± 1.2	8.6 ^cd^ ± 0.5
Rib-eye 7	40.4 ^ab^ ± 4.2	15.8 ^a^ ± 1.2	7.9 ^cd^ ± 0.3
Rib-eye 8	38.0 ^ab^ ± 4.0	18.0 ^ab^ ± 0.4	8.6 ^cd^ ± 0.7
Rib-eye 9	43.2 ^b^ ± 2.8	18.2 ^ab^ ± 1.7	10.3 ^de^ ± 0.9
Rib-eye 10	39.0 ^ab^ ± 2.5	19.9 ^ab^ ± 1.5	10.2 ^de^ ± 0.9
Rib-eye 11	40.1 ^ab^ ± 3.6	18.2 ^ab^ ± 2.2	8.3 ^cd^ ± 1.0
Sirloin 1	31.1 ^a^ ± 2.7	15.4 ^a^ ± 0.9	5.2 ^ab^ ± 0.4
Sirloin 2	44.1 ^b^ ± 0.9	18.1 ^ab^ ± 1.3	9.7 ^cde^ ± 0.5
Sirloin 3	34.8 ^a^ ± 1.9	18.4 ^ab^ ± 1.6	7.6 ^bc^ ± 0.6
*p*-value	0.0016	0.0010	<0.0001

All data are presented as mean ± standard deviation (SD); different lowercase letters (a–e) within the same column indicate that mean values are significantly different (Tukey’s HSD, *p* ≤ 0.05), *p*-values obtained using one-way ANOVA.

**Table 2:** Water activity (a_w_) and pH of the crust and interior of the commercial dry-aged beef steaks from Poland.

Steak Type	Water Activity (a_w_)			pH		
	Crust	Interior	*p*-Value (Crust vs. Interior)	Crust	Interior	*p*-Value (Crust vs. Interior)
Rib-eye 1	0.96 ^aA^ ± 0.02	0.98 ^aA^ ± 0.02	0.4832	5.7 ^aA^ ± 0.2	5.7 ^abA^ ± 0.2	0.8779
Rib-eye 2	0.95 ^aA^ ± 0.03	0.96 ^aA^ ± 0.01	0.6837	6.7 ^bB^ ± 0.2	6.1 ^abA^ ± 0.1	0.0173
Rib-eye 3	0.97 ^aA^ ± 0.02	0.98 ^aA^ ± 0.02	0.5484	5.9 ^aA^ ± 0.2	5.5 ^aA^ ± 0.2	0.2999
Rib-eye 4	0.98 ^aA^ ± 0.02	0.99 ^aA^ ± 0.02	0.9899	6.6 ^bA^ ± 0.3	6.2 ^bA^ ± 0.1	0.0905
Rib-eye 5	0.97 ^aA^ ± 0.02	0.97 ^aA^ ± 0.01	0.3346	5.6 ^aA^ ± 0.2	5.3 ^aA^ ± 0.1	0.2001
Rib-eye 6	0.95 ^aA^ ± 0.03	0.97 ^aA^ ± 0.02	0.9887	5.6 ^aA^ ± 0.2	5.5 ^aA^ ± 0.1	0.5443
Rib-eye 7	0.97 ^aA^ ± 0.03	0.96 ^aA^ ± 0.02	0.6130	5.6 ^aA^ ± 0.2	5.4 ^aA^ ± 0.3	0.5536
Rib-eye 8	0.97 ^aA^ ± 0.03	0.98 ^aA^ ± 0.02	0.1049	5.7 ^aA^ ± 0.2	5.5 ^aA^ ± 0.1	0.3185
Rib-eye 9	0.96 ^aA^ ± 0.03	0.97 ^aA^ ± 0.02	0.9889	5.7 ^aA^ ± 0.2	5.6 ^aA^ ± 0.1	0.5562
Rib-eye 10	0.92 ^aA^ ± 0.02	0.95 ^aA^ ± 0.02	0.7040	5.9 ^aA^ ± 0.1	5.5 ^aA^ ± 0.1	0.1052
Rib-eye 11	0.95 ^aA^ ± 0.01	0.95 ^aA^ ± 0.01	0.6290	5.9 ^aA^ ± 0.2	5.8 ^abA^ ± 0.1	0.4305
Sirloin 1	0.96 ^aA^ ± 0.03	0.97 ^aA^ ± 0.03	0.5733	5.8 ^aA^ ± 0.1	5.8 ^abA^ ± 0.3	0.6922
Sirloin 2	0.96 ^aA^ ± 0.03	0.97 ^aA^ ± 0.02	0.6214	5.7 ^aA^ ± 0.1	5.6 ^aA^ ± 0.2	0.4889
Sirloin 3	0.95 ^aA^ ± 0.01	0.96 ^aA^ ± 0.03	0.5718	5.5 ^aA^ ± 0.1	5.5 ^aA^ ± 0.1	0.6829
*p*-value	0.4323	0.6294		0.0001	0.0023	

All data are presented as mean ± standard deviation (SD); different lowercase letters (a,b) within the same column indicate that mean values are significantly different (Tukey’s HSD, *p* ≤ 0.05), *p*-values were obtained using one-way ANOVA; different uppercase letters (A,B) within the same row indicate significant differences between crust and interior (Student’s *t*-test, *p* ≤ 0.05).

**Table 3:** TBARS values and water-holding capacity (WHC) of the crust and interior of the commercial dry-aged beef steaks from Poland.

Steak Type	TBARS [mg MDA/kg of Product]			Water-Holding Capacity—WHC [cm^2^/g]		
	Crust	Interior	*p*-Value (Crust vs. Interior)	Crust	Interior	*p*-Value (Crust vs. Interior)
Rib-eye 1	2.31 ^eB^ ± 0.13	0.97 ^cA^ ± 0.06	0.0003	1.1 ^aA^ ± 0.2	9.1 ^deB^ ± 1.4	0.0016
Rib-eye 2	1.14 ^dA^ ± 0.05	0.83 ^bcA^ ± 0.10	0.4066	0.0 ^aA^	0.0 ^aA^	1.0000
Rib-eye 3	0.56 ^abA^ ± 0.05	0.41 ^abA^ ± 0.05	0.1639	0.0 ^aA^	7.6 ^cdB^ ± 0.5	0.0193
Rib-eye 4	0.42 ^aA^ ± 0.03	0.37 ^abA^ ± 0.03	0.1583	7.2 ^bA^ ± 0.6	8.6 ^dA^ ± 1.5	0.8883
Rib-eye 5	0.37 ^aB^ ± 0.04	0.25 ^aA^ ± 0.02	0.0151	1.1 ^aA^ ± 0.2	9.6 ^deB^ ± 0.3	0.0027
Rib-eye 6	0.43 ^aA^ ± 0.03	0.39 ^abA^ ± 0.02	0.5484	0.0 ^aA^	0.0 ^aA^	1.0000
Rib-eye 7	0.33 ^aB^ ± 0.01	0.24 ^aA^ ± 0.03	0.0053	0.9 ^aA^ ± 0.1	7.3 ^cdB^ ± 0.8	0.0002
Rib-eye 8	0.50 ^aB^ ± 0.04	0.34 ^aA^ ± 0.05	0.0387	0.0 ^aA^	7.5 ^cdB^ ± 1.4	0.0001
Rib-eye 9	0.52 ^aA^ ± 0.03	0.46 ^abA^ ± 0.02	0.4467	0.0 ^aA^	5.8 ^cdB^ ± 1.1	0.0011
Rib-eye 10	0.57 ^abA^ ± 0.06	0.49 ^abA^ ± 0.03	0.1018	0.0 ^aA^	2.3 ^aB^ ± 0.1	<0.0001
Rib-eye 11	0.45 ^aB^ ± 0.04	0.31 ^abA^ ± 0.04	0.0164	0.0 ^aA^	0.0 ^aA^	1.0000
Sirloin 1	0.79 ^bcA^ ± 0.09	0.64 ^bcA^ ± 0.07	0.1594	7.4 ^bA^ ± 1.1	8.1 ^dA^ ± 0.7	0.3325
Sirloin 2	1.17 ^dB^ ± 0.07	0.35 ^abA^ ± 0.03	0.0005	0.9 ^aA^ ± 0.2	7.0 ^bcdB^ ± 1.1	0.0023
Sirloin 3	0.90 ^cdB^ ± 0.12	0.64 ^abcA^ ± 0.06	0.0209	14.6 ^cA^ ± 0.6	19.1 ^eB^ ± 2.1	0.0272
*p*-value	<0.0001	<0.0001		<0.0001	<0.0001	

All data are presented as mean ± standard deviation (SD); different lowercase letters (a–e) within the same column indicate that mean values are significantly different (Tukey’s HSD, *p* ≤ 0.05), *p*-values were obtained using one-way ANOVA; different uppercase letters (A,B) within the same row indicate significant differences between crust and interior (Student’s *t*-test, *p* ≤ 0.05).

**Table 4:** Basic chemical composition of the crust and interior of the commercial dry-aged beef steaks from Poland.

Steak Type	Protein Content [%]			Water Content [%]			Fat Content [%]		
	Crust	Interior	*p*-Value (Crust vs. Interior)	Crust	Interior	*p*-Value (Crust vs. Interior)	Crust	Interior	*p*-Value (Crust vs. Interior)
Rib-eye 1	24.28 ^cdB^ ± 1.45	18.91 ^abA^ ± 1.24	0.0018	59.36 ^bcdA^ ± 4.02	62.37 ^abcA^ ± 4.48	0.4351	15.34 ^efB^ ± 0.92	13.41 ^cdA^ ± 0.43	0.0301
Rib-eye 2	22.78 ^bcdA^ ± 2.09	19.10 ^abA^ ± 1.06	0.0532	34.80 ^aA^ ± 2.41	56.05 ^aB^ ± 3.93	0.0013	38.18 ^gB^ ± 1.91	22.08 ^fA^ ± 0.38	0.0001
Rib-eye 3	19.77 ^abB^ ± 1.42	16.88 ^aA^ ± 0.74	0.0356	54.29 ^bcA^ ± 4.82	62.94 ^abcA^ ± 4.75	0.0915	23.15 ^gB^ ± 2.21	16.82 ^fA^ ± 1.46	0.0144
Rib-eye 4	20.54 ^abA^ ± 0.89	19.05 ^abA^ ± 1.33	0.1832	59.88 ^bcdA^ ± 4.75	65.74 ^abcdA^ ± 6.27	0.2665	19.86 ^fgB^ ± 1.50	11.09 ^dA^ ± 0.48	0.0006
Rib-eye 5	21.27 ^abcA^ ± 1.33	21.10 ^abcA^ ± 1.06	0.8759	63.44 ^cdeA^ ± 5.19	70.51 ^bcdA^ ± 3.23	0.1157	14.22 ^cdB^ ± 1.07	4.23 ^bA^ ± 0.33	0.0001
Rib-eye 6	19.67 ^abA^ ± 1.09	19.14 ^abA^ ± 1.34	0.6210	60.56 ^bcdA^ ± 3.78	62.67 ^abcA^ ± 4.73	0.5783	17.38 ^fA^ ± 0.18	16.15 ^deA^ ± 1.01	0.1053
Rib-eye 7	22.57 ^bcdA^ ± 1.76	21.40 ^abcA^ ± 1.70	0.4545	61.88 ^cdeA^ ± 4.91	73.15 ^cdA^ ± 5.12	0.0513	14.00 ^cdB^ ± 0.74	1.25 ^aA^ ± 0.11	0.0001
Rib-eye 8	23.64 ^cdA^ ± 1.23	23.20 ^defA^ ± 1.29	0.6889	61.46 ^cdeA^ ± 3.84	69.66 ^bcdB^ ± 1.84	0.0290	11.64 ^cB^ ± 0.61	5.31 ^bA^ ± 0.51	0.0002
Rib-eye 9	24.63 ^cdA^ ± 1.54	22.11 ^cdefA^ ± 1.76	0.1344	55.72 ^bcA^ ± 0.56	58.30 ^abA^ ± 3.08	0.2266	18.00 ^efA^ ± 0.48	16.65 ^fA^ ± 0.88	0.0801
Rib-eye 10	25.17 ^dA^ ± 1.74	23.99 ^efA^ ± 1.50	0.4231	52.35 ^bcA^ ± 4.19	61.99 ^abcA^ ± 5.07	0.0642	21.12 ^fgB^ ± 1.69	13.41 ^eA^ ± 0.97	0.0023
Rib-eye 11	25.42 ^dA^ ± 2.02	23.92 ^efA^ ± 0.83	0.2990	49.50 ^bA^ ± 3.24	60.54 ^abcB^ ± 4.57	0.0270	22.60 ^fgB^ ± 1.38	13.50 ^eA^ ± 0.68	0.0005
Sirloin 1	21.73 ^abcA^ ± 1.85	19.90 ^abA^ ± 1.82	0.2891	72.47 ^eA^ ± 4.41	75.63 ^dA^ ± 6.72	0.5337	1.66 ^aB^ ± 0.09	1.07 ^aA^ ± 0.09	0.0012
Sirloin 2	17.79 ^aA^ ± 0.94	17.67 ^abA^ ± 0.31	0.8354	63.85 ^cdeA^ ± 4.19	69.77 ^bcdA^ ± 2.51	0.1037	14.58 ^cdeB^ ± 0.91	8.46 ^cA^ ± 0.77	0.0009
Sirloin 3	25.68 ^dA^ ± 1.43	24.17 ^fA^ ± 1.89	0.3313	64.99 ^deA^ ± 5.32	70.41 ^bcdA^ ± 2.44	0.1840	6.53 ^bB^ ± 0.53	3.41 ^bA^ ± 0.11	0.0006
*p*-value	<0.0001	<0.0001		<0.0001	<0.0001		<0.0001	<0.0001	

All data are presented as mean ± standard deviation (SD); different lowercase letters (a–g) within the same column indicate that mean values are significantly different (Tukey’s HSD, *p* ≤ 0.05), *p*-values were obtained using one-way ANOVA; different uppercase letters (A,B) within the same row indicate significant differences between crust and interior (Student’s *t*-test, *p* ≤ 0.05).

**Table 5:** Cooking yield and texture parameters of the commercial dry-aged beef steaks from Poland.

Steak Type	Cooking Yield [%]	Warner-Bratzler Shear Force—WBSF [N]	Texture Profile Analysis—TPA			
			Cohesiveness [−]	Springiness [−]	Hardness [N]	Chewiness [N]
Rib-eye 1	83.3 ^a^ ± 7.3	52.4 ^abc^ ± 8.8	0.4 ^a^ ± 0.1	0.5 ^a^ ± 0.1	12.5 ^a^ ± 3.8	3.2 ^a^ ± 0.8
Rib-eye 2	88.4 ^a^ ± 8.5	59.4 ^abc^ ± 16.6	0.6 ^a^ ± 0.1	0.6 ^a^ ± 0.1	57.7 ^c^ ± 11.8	18.9 ^ef^ ± 5.5
Rib-eye 3	85.7 ^a^ ± 9.2	49.9 ^abc^ ± 11.2	0.5 ^a^ ± 0.1	0.6 ^a^ ± 0.1	43.7 ^abc^ ± 7.3	13.5 ^cdef^ ± 3.1
Rib-eye 4	88.7 ^a^ ± 5.3	49.6 ^abc^ ± 14.3	0.4 ^a^ ± 0.1	0.5 ^a^ ± 0.1	19.0 ^a^ ± 3.9	5.5 ^ab^ ± 0.6
Rib-eye 5	78.2 ^a^ ± 5.5	70.1 ^bc^ ± 16.4	0.4 ^a^ ± 0.1	0.5 ^a^ ± 0.1	26.0 ^ab^ ± 5.4	6.7 ^abc^ ± 0.9
Rib-eye 6	80.1 ^a^ ± 4.0	49.7 ^abc^ ± 12.4	0.4 ^a^ ± 0.1	0.4 ^a^ ± 0.1	27.6 ^ab^ ± 5.8	4.0 ^ab^ ± 0.4
Rib-eye 7	76.2 ^a^ ± 11.7	59.5 ^abc^ ± 18.5	0.5 ^a^ ± 0.1	0.5 ^a^ ± 0.1	19.6 ^ab^ ± 3.1	4.5 ^ab^ ± 1.2
Rib-eye 8	81.9 ^a^ ± 6.3	28.1 ^ab^ ± 7.6	0.6 ^a^ ± 0.1	0.5 ^a^ ± 0.1	60.0 ^c^ ± 13.3	19.1 ^f^ ± 1.9
Rib-eye 9	87.9 ^a^ ± 11.7	80.6 ^c^ ± 17.5	0.6 ^a^ ± 0.2	0.6 ^a^ ± 0.2	17.5 ^a^ ± 2.5	5.8 ^ab^ ± 0.6
Rib-eye 10	74.2 ^a^ ± 7.6	53.2 ^abc^ ± 15.7	0.6 ^a^ ± 0.2	0.5 ^a^ ± 0.1	28.4 ^ab^ ± 3.6	8.6 ^abcd^ ± 0.6
Rib-eye 11	73.8 ^a^ ± 2.3	24.3 ^a^ ± 5.0	0.4 ^a^ ± 0.1	0.5 ^a^ ± 0.1	97.7 ^d^ ± 12.6	16.3 ^def^ ± 5.9
Sirloin 1	77.1 ^a^ ± 12.5	74.0 ^c^ ± 21.2	0.5 ^a^ ± 0.1	0.5 ^a^ ± 0.1	40.3 ^abc^ ± 10.0	10.7 ^abcde^ ± 2.5
Sirloin 2	68.2 ^a^ ± 12.0	44.4 ^abc^ ± 14.0	0.4 ^a^ ± 0.1	0.5 ^a^ ± 0.1	12.8 ^a^ ± 2.3	2.7 ^a^ ± 0.2
Sirloin 3	70.0 ^a^ ± 11.6	51.7 ^abc^ ± 15.3	0.5 ^a^ ± 0.1	0.6 ^a^ ± 0.2	48.6 ^bc^ ± 15.6	14.3 ^cdef^ ± 1.8
*p*-value	0.1717	0.0039	0.2407	0.9517	<0.0001	<0.0001

All data are presented as mean ± standard deviation (SD); different lowercase letters (a–f) within the same column indicate that mean values are significantly different (Tukey’s HSD, *p* ≤ 0.05), *p*-values were obtained using one-way ANOVA.

**Table 6:** Total plate count, psychrotrophic bacteria count, and lactic acid bacteria of the crust and interior of the commercial dry-aged beef steaks from Poland.

Steak Type	Total Plate Count (TPC) [log cfu/g]			Psychrotrophic Bacteria Count (PBC) [log cfu/g]			Lactic Acid Bacteria (LAB) [log cfu/g]		
	Crust	Interior	*p*-Value (Crust vs. Interior)	Crust	Interior	*p*-Value (Crust vs. Interior)	Crust	Interior	*p*-Value (Crust vs. Interior)
Rib-eye 1	6.21 ^aB^ ± 0.10	5.73 ^aA^ ± 0.06	0.0014	5.40 ^aB^ ± 0.01	5.10 ^abA^ ± 0.01	0.0012	3.80 ^abB^ ± 0.01	3.27 ^abA^ ± 0.06	<0.0001
Rib-eye 2	6.07 ^aA^ ± 0.06	6.07 ^aA^ ± 0.06	1.0000	5.50 ^aB^ ± 0.10	5.03 ^abA^ ± 0.06	0.0015	4.27 ^cdB^ ± 0.06	3.97 ^abA^ ± 0.06	0.0007
Rib-eye 3	6.17 ^aB^ ± 0.06	5.97 ^aA^ ± 0.10	0.0044	5.47 ^aB^ ± 0.06	5.33 ^abA^ ± 0.05	0.0212	3.80 ^abB^ ± 0.01	3.30 ^abA^ ± 0.07	<0.0001
Rib-eye 4	7.90 ^eA^ ± 0.01	7.87 ^eA^ ± 0.07	0.2336	7.37 ^cB^ ± 0.06	7.13 ^eA^ ± 0.06	0.0016	4.43 ^dB^ ± 0.05	4.00 ^dA^ ± 0.04	<0.0001
Rib-eye 5	6.67 ^aB^ ± 0.06	6.00 ^aA^ ± 0.01	0.0003	6.63 ^aB^ ± 0.06	5.73 ^abA^ ± 0.07	0.0003	4.37 ^dA^ ± 0.06	4.40 ^fA^ ± 0.01	1.0000
Rib-eye 6	7.80 ^dB^ ± 0.01	7.37 ^dA^ ± 0.07	0.0002	7.07 ^bB^ ± 0.06	6.97 ^dA^ ± 0.06	0.0189	4.77 ^eB^ ± 0.10	4.70 ^hA^ ± 0.02	0.0138
Rib-eye 7	6.47 ^aB^ ± 0.06	5.47 ^aA^ ± 0.06	0.0002	6.33 ^aA^ ± 0.07	6.33 ^bcA^ ± 0.08	0.3341	4.23 ^dB^ ± 0.06	4.10 ^dA^ ± 0.06	0.0012
Rib-eye 8	7.27 ^bB^ ± 0.06	6.93 ^cA^ ± 0.07	0.0020	6.57 ^aB^ ± 0.06	5.90 ^aB^ ± 0.10	0.0006	4.90 ^fB^ ± 0.01	4.43 ^gA^ ± 0.08	0.0002
Rib-eye 9	7.83 ^dB^ ± 0.06	6.17 ^abA^ ± 0.06	0.0003	7.70 ^eB^ ± 0.10	6.00 ^abcA^ ± 0.01	0.0002	4.37 ^dB^ ± 0.06	2.77 ^aA^ ± 0.10	0.0001
Rib-eye 10	5.30 ^aA^ ± 0.01	5.30 ^aA^ ± 0.01	1.0000	5.63 ^aB^ ± 0.06	5.00 ^aA^ ± 0.10	0.0006	3.30 ^aB^ ± 0.01	2.30 ^aA^ ± 0.01	0.0006
Rib-eye 11	7.47 ^bcB^ ± 0.06	7.03 ^cA^ ± 0.06	0.0001	7.63 ^deB^ ± 0.06	7.07 ^eA^ ± 0.06	0.0001	4.30 ^cdB^ ± 0.01	3.63 ^bcA^ ± 0.09	0.0001
Sirloin 1	6.87 ^abB^ ± 0.06	6.30 ^abA^ ± 0.01	0.0004	5.33 ^aA^ ± 0.06	5.13 ^abA^ ± 0.05	0.0339	3.47 ^abA^ ± 0.08	3.43 ^abA^ ± 0.10	0.3402
Sirloin 2	5.37 ^aB^ ± 0.06	5.07 ^aA^ ± 0.06	0.0023	5.13 ^aB^ ± 0.05	4.90 ^aA^ ± 0.01	0.0019	3.53 ^abB^ ± 0.06	3.30 ^abA^ ± 0.01	0.0002
Sirloin 3	7.53 ^cB^ ± 0.06	6.37 ^abA^ ± 0.06	<0.0001	7.60 ^dB^ ± 0.01	6.43 ^cA^ ± 0.08	<0.0001	5.00 ^gB^ ± 0.01	3.80 ^cA^ ± 0.01	<0.0001
*p*-value	<0.0001	<0.0001		<0.0001	<0.0001		<0.0001	<0.0001	

All data are presented as mean ± standard deviation (SD); different lowercase letters (a–h) within the same column indicate that mean values are significantly different (Tukey’s HSD, *p* ≤ 0.05), *p*-values were obtained using one-way ANOVA; different uppercase letters (A,B) within the same row indicate significant differences between crust and interior (Student’s *t*-test, *p* ≤ 0.05).

**Table 7:** Enterobacteriaceae, yeasts and moulds (YAMs), *Brochothrix thermosphacta*, and *Pseudomonas* spp. of the crust and interior of the commercial dry-aged beef steaks from Poland.

Steak Type	Enterobacteriaceae [log cfu/g]			Yeasts and Moulds (YAMs) [log cfu/g]			*Brochothrix thermosphacta* [log cfu/g]			*Pseudomonas* spp. [log cfu/g]		
	Crust	Interior	*p*-Value (Crust vs. Interior)	Crust	Interior	*p*-Value (Crust vs. Interior)	Crust	Interior	*p*-Value (Crust vs. Interior)	Crust	Interior	*p*-Value (Crust vs. Interior)
Rib-eye 1	2.10 ^aB^ ± 0.01	<1.00	<0.0001	3.83 ^bcB^ ± 0.06	2.70 ^bcA^ ± 0.01	<0.0001	6.07 ^abB^ ± 0.06	4.67 ^aA^ ± 0.09	0.0007	6.03 ^aB^ ± 0.11	5.60 ^aA^ ± 0.08	0.0001
Rib-eye 2	3.60^cdB^ ± 0.01	3.17 ^bA^ ± 0.06	0.0001	4.30 ^eB^ ± 0.01	<2.00	<0.0001	6.20 ^abcB^ ± 0.01	5.97 ^aA^ ± 0.11	0.0058	6.00 ^aB^ ± 0.01	5.63 ^aA^ ± 0.08	0.0004
Rib-eye 3	3.50 ^cdB^ ± 0.01	2.90 ^abA^ ± 0.01	<0.0001	4.07 ^dB^ ± 0.09	3.70 ^fA^ ± 0.01	0.0003	6.50 ^cdeB^ ± 0.01	5.93 ^aA^ ± 0.10	<0.0001	5.87 ^aA^ ± 0.09	5.77 ^aA^ ± 0.07	0.0664
Rib-eye 4	3.63 ^dB^ ± 0.06	3.07 ^bA^ ± 0.08	0.0001	4.53 ^fB^ ± 0.08	3.80 ^gA^ ± 0.01	<0.0001	7.40 ^fB^ ± 0.01	7.13 ^bA^ ± 0.10	0.0003	7.57 ^cB^ ± 0.06	6.67 ^bA^ ± 0.06	<0.0001
Rib-eye 5	4.20 ^eB^ ± 0.01	3.30 ^cA^ ± 0.01	<0.0001	2.50 ^aB^ ± 0.01	2.30 ^abA^ ± 0.01	0.0001	4.97 ^aB^ ± 0.06	4.90 ^aA^ ± 0.01	0.0583	6.40 ^aB^ ± 0.07	5.43 ^aA^ ± 0.06	0.0003
Rib-eye 6	4.27 ^fB^ ± 0.08	3.70 ^fA^ ± 0.01	0.0001	4.90 ^gB^ ± 0.01	3.67 ^fA^ ± 0.08	<0.0001	6.53 ^dB^ ± 0.07	5.57 ^aA^ ± 0.08	0.0001	7.77 ^dB^ ± 0.08	7.33 ^dA^ ± 0.07	0.0012
Rib-eye 7	3.50 ^cdB^ ± 0.01	3.00 ^bA^ ± 0.01	0.0001	3.07 ^aB^ ± 0.09	<2.00	<0.0001	4.00 ^aA^ ± 0.01	4.03 ^aA^ ± 0.08	1.0000	5.60 ^aB^ ± 0.06	5.10 ^aA^ ± 0.01	0.0008
Rib-eye 8	2.90 ^abB^ ± 0.01	1.97 ^aA^ ± 0.06	<0.0001	2.70 ^ab^ ± 0.01	2.60 ^abcA^ ± 0.01	0.0170	6.30 ^bcdB^ ± 0.01	4.30 ^aA^ ± 0.01	<0.0001	7.20 ^bB^ ± 0.01	7.07 ^cA^ ± 0.0	0.0015
Rib-eye 9	3.63 ^cdB^ ± 0.07	3.30 ^bA^ ± 0.01	0.0001	4.03 ^cdB^ ± 0.05	2.07 ^abA^ ± 0.07	0.0006	6.53 ^cdB^ ± 0.08	4.80 ^aA^ ± 0.01	<0.0001	7.93 ^eB^ ± 0.08	5.90 ^aA^ ± 0.01	0.0002
Rib-eye 10	2.43 ^aB^ ± 0.06	2.10 ^aA^ ± 0.01	0.0002	3.17 ^aB^ ± 0.07	3.07 ^dA^ ± 0.05	0.0232	4.50 ^aA^ ± 0.01	4.47 ^aA^ ± 0.07	1.0000	5.13 ^aB^ ± 0.08	4.73 ^aA^ ± 0.07	0.0019
Rib-eye 11	<1.00	<1.00	1.0000	3.40 ^abB^ ± 0.01	3.23 ^eA^ ± 0.06	0.0014	4.70 ^aB^ ± 0.01	4.47 ^aA^ ± 0.06	0.0041	4.47 ^aB^ ± 0.06	3.97 ^aA^ ± 0.06	0.0007
Sirloin 1	4.20 ^dB^ ± 0.01	3.50 ^bA^ ± 0.01	<0.0001	3.43 ^abB^ ± 0.06	2.87 ^cA^ ± 0.09	<0.0001	6.00 ^abB^ ± 0.01	5.73 ^aA^ ± 0.06	0.0003	5.83 ^aB^ ± 0.07	5.67 ^aA^ ± 0.11	0.0301
Sirloin 2	2.17 ^aB^ ± 0.08	2.10 ^aA^ ± 0.01	0.0308	3.30 ^aB^ ± 0.01	<2.00	<0.0001	4.43 ^aB^ ± 0.08	3.67 ^aA^ ± 0.08	0.0002	5.60 ^aB^ ± 0.06	5.13 ^aA^ ± 0.10	0.0001
Sirloin 3	3.13 ^abB^ ± 0.06	2.33 ^aA^ ± 0.05	<0.0001	4.40 ^dB^ ± 0.01	2.83 ^cA^ ± 0.06	<0.0001	7.23 ^eB^ ± 0.06	5.20 ^aA^ ± 0.01	<0.0001	7.93 ^eB^ ± 0.07	5.90 ^aA^ ± 0.01	0.0001
*p*-value	<0.0001	<0.0001		<0.0001	<0.0001		<0.0001	<0.0001		<0.0001	<0.0001	

All data are presented as mean ± standard deviation (SD); different lowercase letters (a–f) within the same column indicate that mean values are significantly different (Tukey’s HSD, *p* ≤ 0.05), *p*-values were obtained using one-way ANOVA; different uppercase letters (A,B) within the same row indicate significant differences between crust and interior (Student’s *t*-test, *p* ≤ 0.05).

**Table 8:** Free amino acids: histidine, lysine, ornithine, and tyrosine content of the crust and interior of the commercial dry-aged beef steaks from Poland.

Steak Type	Histidine [mg/kg]			Lysine [mg/kg]			Ornithine [mg/kg]			Tyrosine [mg/kg]		
	Crust	Interior	*p*-Value (Crust vs. Interior)	Crust	Interior	*p*-Value (Crust vs. Interior)	Crust	Interior	*p*-Value (Crust vs. Interior)	Crust	Interior	*p*-Value (Crust vs. Interior)
Rib-eye 1	83.9 ^abcA^ ± 13.6	100.3 ^bcA^ ± 14.8	0.2311	548.6 ^cdB^ ± 166.4	271.5 ^abcA^ ± 33.2	0.0475	228.8 ^cdA^ ± 26.0	221.2 ^cdA^ ± 49.9	0.8259	9.8 ^aB^ ± 3.4	3.9 ^aA^ ± 0.8	0.0441
Rib-eye 2	161.3 ^dA^ ± 18.6	207.8 ^efA^ ± 43.8	0.1662	932.6 ^fA^ ± 85.2	1122.6 ^eA^ ± 208.7	0.2181	382.9 ^eA^ ± 53.7	364.3 ^eA^ ± 65.4	0.7225	145.2 ^cB^ ± 22.7	55.8 ^bA^ ± 9.3	0.0032
Rib-eye 3	120.3 ^bcA^ ± 15.3	170.5 ^dB^ ± 22.4	0.0327	73.8 ^aB^ ± 13.7	29.2 ^aA^ ± 4.3	0.0058	164.7 ^bcA^ ± 35.8	105.5 ^bcA^ ± 13.8	0.0557	7.7 ^aB^ ± 1.6	3.6 ^aA^ ± 0.8	0.0161
Rib-eye 4	37.3 ^aA^ ± 4.1	43.7 ^aA^ ± 7.5	0.2639	207.2 ^abA^ ± 34.1	221.1 ^abcA^ ± 26.2	0.6062	205.9 ^cA^ ± 33.4	188.3 ^cA^ ± 29.7	0.5317	4.2 ^aA^ ± 0.4	5.9 ^aB^ ± 1.0	0.0490
Rib-eye 5	50.9 ^abA^ ± 8.0	59.7 ^abA^ ± 5.7	0.1926	174.6 ^abA^ ± 41.2	247.7 ^abcA^ ± 41.4	0.09645	39.9 ^aA^ ± 4.4	36.1 ^bA^ ± 3.4	0.3115	7.9 ^aA^ ± 1.2	8.9 ^aA^ ± 1.5	0.3939
Rib-eye 6	52.5 ^abA^ ± 5.0	72.4 ^abB^ ± 8.3	0.0237	68.1 ^aA^ ± 9.3	86.1 ^aA^ ± 15.4	0.1588	80.4 ^abB^ ± 13.1	45.5 ^bA^ ± 6.0	0.0137	3.7 ^aA^ ± 0.5	7.6 ^aB^ ± 1.9	0.0265
Rib-eye 7	38.0 ^aA^ ± 7.2	48.6 ^aA^ ± 5.5	0.1120	140.5 ^aA^ ± 24.9	207.5 ^abB^ ± 24.2	0.0288	74.1 ^abB^ ± 9.1	33.7 ^bA^ ± 3.5	0.0020	3.5 ^aA^ ± 0.6	10.7 ^aB^ ± 3.1	0.0162
Rib-eye 8	161.7 ^dA^ ± 18.4	172.9 ^efA^ ± 33.9	0.6409	687.2 ^deA^ ± 120.1	737.7 ^dA^ ± 183.2	0.7099	102.9 ^abB^ ± 11.6	41.2 ^bA^ ± 4.3	0.0009	7.4 ^aA^ ± 2.4	12.8 ^aA^ ± 4.9	0.1657
Rib-eye 9	74.5 ^abA^ ± 16.8	81.5 ^abcA^ ± 7.1	0.5396	189.0 ^abA^ ± 36.4	170.8 ^aA^ ± 48.2	0.6277	112.1 ^abA^ ± 12.6	83.6 ^bcA^ ± 16.2	0.0744	13.4 ^aB^ ± 2.6	6.9 ^aA^ ± 0.7	0.0141
Rib-eye 10	119.3 ^bcA^ ± 16.7	141.1 ^cdeA^ ± 21.7	0.2406	384.1 ^bcA^ ± 67.2	490.8 ^cdA^ ± 153.3	0.3313	153.8 ^bcB^ ± 21.8	55.1 ^bA^ ± 6.8	0.0017	14.8 ^aA^ ± 2.7	56.6 ^bB^ ± 12.9	0.0053
Rib-eye 11	150.1 ^dA^ ± 21.3	250.8 ^fB^ ± 44.1	0.0236	825.4 ^efB^ ± 107.1	482.4 ^bcdA^ ± 93.6	0.0139	104.8 ^abB^ ± 22.8	41.0 ^bA^ ± 7.7	0.0100	106.0 ^bA^ ± 18.9	73.0 ^bA^ ± 20.9	0.1125
Sirloin 1	88.2 ^abcA^ ± 13.3	95.3 ^abcdA^ ± 16.8	0.5952	24.1 ^aA^ ± 6.6	23.0^aA^ ± 5.0	0.8277	135.6 ^bcA^ ± 27.4	107.3 ^bcA^ ± 9.8	0.1676	2.3 ^aA^ ± 0.8	2.4 ^aA^ ± 0.4	0.9649
Sirloin 2	88.5 ^abcA^ ± 14.4	119.6 ^bcdeA^ ± 16.7	0.0707	50.9 ^aA^ ± 8.8	228.4^abcB^ ± 25.7	0.0003	209.9 ^cB^ ± 25.71	121.5 ^cA^ ± 26.5	0.0142	2.9 ^aA^ ± 0.7	4.2 ^aB^ ± 0.4	0.0382
Sirloin 3	81.0 ^abcA^ ± 8.0	92.7 ^abcdA^ ± 18.0	0.3619	199.9 ^abA^ ± 50.0	255.3 ^abcA^ ± 42.4	0.2170	80.7 ^abB^ ± 9.1	41.0 ^bA^ ± 5.0	0.0027	5.2 ^aA^ ± 1.8	14.7 ^aB^ ± 4.9	0.0344
*p*-value	<0.0001	<0.0001		<0.0001	<0.0001		<0.0001	<0.0001		<0.0001	<0.0001	

All data are presented as mean ± standard deviation (SD); different lowercase letters (a–e) within the same column indicate that mean values are significantly different (Tukey’s HSD, *p* ≤ 0.05), *p*-values were obtained using one-way ANOVA; different uppercase letters (A,B) within the same row indicate significant differences between crust and interior (Student’s *t*-test, *p* ≤ 0.05).

**Table 9:** Biogenic amines: histamine, cadaverine, putrescine and tyramine content, and biogenic amines index (BAI) of the crust and interior of commercial dry-aged beef steaks from Poland.

Steak Type	Histamine (HIS) [mg/kg]			Cadaverine (CAD) [mg/kg]			Putrescine (PUT) [mg/kg]			Tyramine (TYM) [mg/kg]			BAI [mg/kg]		
	Crust	Interior	*p*-Value (Crust vs. Interior)	Crust	Interior	*p*-Value (Crust vs. Interior)	Crust	Interior	*p*-Value (Crust vs. Interior)	Crust	Interior	*p*-Value (Crust vs. Interior)	Crust	Interior	*p*-Value (Crust vs. Interior)
Rib-eye 1	3.8 ^abA^ ± 1.3	2.3 ^aA^ ± 0.5	0.1306	70.0 ^abcB^ ± 21.2	38.3 ^abA^ ± 7.3	0.0409	10.6 ^aB^ ± 1.5	7.3 ^aA^ ± 1.2	0.0402	184.9 ^cdeA^ ± 19.7	162.7 ^bcdA^ ± 9.9	0.1558	269.3 ^bcA^ ± 40.6	210.6 ^bcA^ ± 18.6	0.6692
Rib-eye 2	9.5 ^cA^ ± 3.1	5.6 ^bA^ ± 0.8	0.1015	45.7 ^abA^ ± 14.8	21.1 ^abA^ ± 8.4	0.0665	36.1 ^bA^ ± 7.0	25.6 ^bA^ ± 4.1	0.0883	319.0 ^fB^ ± 49.9	151.3 ^bcdA^ ± 15.8	0.0051	410.3 ^bcB^ ± 74.5	203.6 bc^A^ ± 28.6	0.0396
Rib-eye 3	2.5 ^aA^ ± 0.7	2.3 ^aA^ ± 1.0	0.7884	453.3 ^dB^ ± 108.4	263.7 ^dA^ ± 69.6	0.0415	149.2 ^dB^ ± 28.1	34.2 ^bcA^ ± 7.8	0.0024	257.8 ^efA^ ± 35.3	210.1 ^deA^ ± 12.5	0.0919	862.6 ^eA^ ± 238.0	510.5 ^eA^ ± 87.6	0.0741
Rib-eye 4	1.9 ^aA^ ± 0.3	1.5 ^aA^ ± 0.3	0.2089	17.9 ^abB^ ± 4.2	1.8 ^aA^ ± 0.5	0.0026	24.7 ^abB^ ± 4.1	1.7 ^aA^ ± 0.3	0.0006	128.5 ^abcA^ ± 11.1	125.6 ^abcA^ ± 13.7	0.7903	173.0 ^abA^ ± 18.1	130.6 ^abA^ ± 14.8	0.0577
Rib-eye 5	1.7 ^aA^ ± 0.3	1.1 ^aA^ ± 0.2	0.0610	27.8 ^abA^ ± 7.1	20.3 ^abA^ ± 5.4	0.2197	2.1 ^aA^ ± 0.4	2.0 ^aA^ ± 0.2	0.7410	172.3 ^cdeA^ ± 19.6	144.1 ^abcA^ ± 17.4	0.1353	203.9 ^abA^ ± 25.0	167.5 ^bcA^ ± 23.0	0.3519
Rib-eye 6	14.6 ^dB^ ± 4.2	5.5 ^bA^ ± 0.9	0.0210	106.8 ^abcA^ ± 29.7	98.7 ^bcA^ ± 43.0	0.8016	41.6 ^bA^ ± 6.6	34.0 ^bcA^ ± 9.8	0.3261	143.6 ^abcA^ ± 13.4	129.3 ^abcA^ ± 10.1	0.2138	306.6 ^bcA^ ± 46.8	267.5 ^cdA^ ± 61.4	0.4300
Rib-eye 7	2.1 ^aA^ ± 0.5	1.6 ^aA^ ± 0.5	0.3006	20.5 ^abA^ ± 9.3	9.5 ^abA^ ± 2.9	0.1222	2.6 ^aA^ ± 0.3	2.5 ^aA^ ± 0.6	0.8188	125.4 ^abcA^ ± 6.6	109.4 ^abA^ ± 10.2	0.0846	150.6 ^abA^ ± 14.0	123.0 ^aA^ ± 10.5	0.6651
Rib-eye 8	2.5 ^aA^ ± 0.3	2.1 ^aA^ ± 0.5	0.3252	21.2 ^abA^ ± 8.3	10.7 ^abA^ ± 2.6	0.1051	1.6 ^aA^ ± 0.4	1.1 ^aA^ ± 0.1	0.0824	232.1 ^efA^ ± 35.7	219.0 ^eA^ ± 16.6	0.5952	257.4 ^bA^ ± 44.5	232.9 ^bcdA^ ± 19.4	0.9503
Rib-eye 9	2.5 ^aA^ ± 0.5	2.1 ^aA^ ± 0.3	0.3293	28.2 ^abA^ ± 7.9	20.4 ^abA^ ± 8.1	0.2980	4.0 ^aA^ ± 0.8	2.7 ^aA^ ± 0.4	0.0653	113.0 ^abcA^ ± 16.4	94.4 ^aA^ ± 12.0	0.1856	147.7 ^aA^ ± 20.2	119.6 ^aA^ ± 20.8	0.1688
Rib-eye 10	2.2 ^aA^ ± 0.5	2.0 ^aA^ ± 0.5	0.6363	14.8 ^abB^ ± 2.6	4.0 ^aA^ ± 1.3	0.0029	2.1 ^aB^ ± 0.2	1.5 ^aA^ ± 0.1	0.0189	179.3 ^cdeB^ ± 20.3	139.5 ^abcdeA^ ± 6.8	0.0323	198.4 ^abA^ ± 22.8	147.0 ^abA^ ± 8.7	0.1122
Rib-eye 11	7.8 ^bcA^ ± 2.3	5.8 ^bA^ ± 1.6	0.2815	2.5 ^aB^ ± 0.8	0.8 ^aA^ ± 0.2	0.0244	10.2 ^aB^ ± 1.0	4.4 ^aA^ ± 0.7	0.0012	163.9 ^cdeB^ ± 8.6	132.8 ^abcA^ ± 16.9	0.0470	184.4 ^abB^ ± 11.9	143.8 ^abA^ ± 19.4	0.0363
Sirloin 1	2.4 ^aB^ ± 0.5	1.2 ^aA^ ± 0.3	0.0215	286.5 ^cdA^ ± 111.0	244.3 ^dA^ ± 68.7	0.6053	82.1 ^cB^ ± 9.7	44.9 ^cA^ ± 9.9	0.0097	180.4 ^cdeA^ ± 15.2	179.7 ^cdeA^ ± 12.7	0.9540	551.4 ^dA^ ± 121.0	470.1 ^deA^ ± 88.2	0.4003
Sirloin 2	1.6 ^aA^ ± 0.6	1.2 ^aA^ ± 0.2	0.3218	185.2 ^bcA^ ± 71.0	135.7 ^cA^ ± 42.7	0.3595	40.8 ^bB^ ± 5.2	2.8 ^aA^ ± 0.4	0.0002	219.3 ^deB^ ± 11.9	165.9 ^cdeA^ ± 23.2	0.0240	446.9 ^cdA^ ± 84.9	305.6 ^cdA^ ± 64.4	0.6118
Sirloin 3	2.3 ^aA^ ± 0.8	1.9 ^aA^ ± 0.6	0.5104	6.1 ^abB^ ± 1.3	2.3 ^aA^ ± 1.0	0.0163	1.5 ^aA^ ± 0.2	1.2 ^aA^ ± 0.3	0.2660	111.3 ^abA^ ± 16.7	94.3 ^aA^ ± 12.9	0.2360	121.2 ^aA^ ± 18.4	99.7 ^aA^ ± 14.7	0.4074
*p*-value	<0.0001	<0.0001		<0.0001	<0.0001		<0.0001	<0.0001		<0.0001	<0.0001		<0.0001	<0.0001	

All data are presented as mean ± standard deviation (SD); different lowercase letters (a–f) within the same column indicate that mean values are significantly different (Tukey’s HSD, *p* ≤ 0.05), *p*-values were obtained using one-way ANOVA; different uppercase letters (A,B) within the same row indicate significant differences between crust and interior (Student’s *t*-test, *p* ≤ 0.05).

## Data Availability

The experimental data are available in an open repository at the following link: https://doi.org/10.18150/PPDNDC.

## Supplementary Materials

The following supporting information can be downloaded at https://www.mdpi.com/article/10.3390/foods15132345/s1, Table S1: The *m*/*z* values of dansyl derivatives’ ions used for free amino acids and biogenic amines quantification; Table S2. Molecular identification of fungal isolates based on ITS rDNA sequence analysis using GenBank and UNITE databases; Table S3: Free amino acid profile of the crust and interior of commercial dry-aged beef steaks from Poland (not included in Table 8); Table S4: Free amino acid profile of the crust and interior of commercial dry-aged beef steaks from Poland (not included in Table 8); Table S5: Free amino acid profile of the crust and interior of commercial dry-aged beef steaks from Poland (not included in Table 8); Table S6: Biogenic amine (BA) profile of the crust and interior of commercial dry-aged beef steaks from Poland (not included in Table 9); Figure S1. Morphological and microscopic characteristics of mould isolates from dry-aged beef. Colony appearance (A) and microscope image (B) of *Penicillium palitans*; colony appearance (C) and microscope image (D) of *Penicillium solitum*; colony appearance (E) and microscope image (F) of *Iprex laceratus*; colony appearance (G) and microscope image (H) of *Aureobasidium melanogenum*; colony appearance (I) and microscope image (J) of *Phoma herbarum*.
