# Supplementary material for: Quality Assessment of Commercial Dry-Aged Beef Produced in Poland
Source: Foods. 2026 Jul 2;15(13):2345. doi: 10.3390/foods15132345 (PMC13362366; doi:10.3390/foods15132345)
Supplement: Supplementary file 1 [file foods-15-02345-s001.zip › foods-4345225-supplementary.pdf]

Table S1. The *m/z* values of dansyl derivatives' ions used for free amino acids and biogenic amines quantification.

Dansyl derivative	*m/z*
Agmatine	364.18
2-phenylethylamine	355.15
Putrescine	555.21
Cadaverine	569.23
Histamine	345.14, 578.19
Tryptamine	394.16
Tyramine	604.19
Spermidine	845.32
Spermine	1135.42
Asparagine	366.11
Glutamine	380.13
Arginine	408.17
Serine	339.10
Aspartic acid	367.10
Glutamic acid	381.11
Threonine	353.12
Glycine	309.09
Tryptophan	438.15
Alanine	323.11
Proline	349.12
Valine	351.14
Methionine	383.11
Phenylalanine	399.14
Leucine/isoleucine	365.15
Ornithine	599.20
Lysine	613.22
Histidine	389.13, 622.18
Tyrosine	648.18
1,7-diaminoheptane	597.26

Table S2. Molecular identification of fungal isolates based on ITS rDNA sequence analysis using GenBank and UNITE databases

Isolate	Closest GenBank match (Accession No.)	Closest UNITE match (SH accession)	Identity (%)	Query coverage (%)	E-value	Closest match
F1	OP162804.1	SH1801174.10FU	100.0	100	0.0	*Penicillium palitans*
	MF803944.1	SH1801174.10FU	100.0	100	0.0	
F2	OW982516.1	SH1801174.10FU	100.0	100	0.0	*Penicillium solitum*
	OW986444.1	SH1801174.10FU	100.0	100	0.0	
	OW988404.1	SH1801174.10FU	100.0	100	0.0	
F3	KC311476.1	SH1672908.10FU	100.0	100	0.0	*Phoma herbarum*
	MF120206.1	SH1672908.10FU	100.0	100	0.0	
F4	ON074970	SH1778424.10FU	100.0	100	0.0	*Aureobasidium*
	ON074942	SH1778424.10FU	100.0	100	0.0	*melanogenum*
F5	PV342503	SH1486907.10FU	99.43	100	0.0	*Irpex lacerates*
	PV342506	SH1486907.10FU	99.29	100	0.0	
	PV342505	SH1486907.10FU	99.15	100	0.0	
F6	MH859070	SH1798015.10FU	100	100	0.0	*Mucor flavus*
	MF615061	SH1798015.10FU	100	100	0.0	
F7	OW987295	SH1798004.10FU	100.0	100	0.0	*Thamnidium elegans*
	OW982730	SH1798004.10FU	100.0	100	0.0	

Table S3. Free amino acids profile of the crust and interior of commercial dry-aged beef steaks from Poland (not included in Table 8).

Steak type	Asparagine [mg/kg]			Glutamine [mg/kg]			Serine [mg/kg]			Aspartic acid [mk/kg]			Phenylalanine [mg/kg]		
	**crust**	**interior**	**p – value (crust vs. interior)**	**crust**	**interior**	**p – value (crust vs. interior)**	**crust**	**interior**	**p – value (crust vs. interior)**	**crust**	**interior**	**p – value (crust vs. interior)**	**crust**	**interior**	**p – value (crust vs. interior)**
Rib-eye 1	$49.0^{abcdA}\pm6.8$	$98.4b^{cdeB}\pm26.1$	0.0338	$13.1^{aA}\pm5.1$	$260.5^{abB}\pm90.3$	0.0090	$331.5^{abcA}\pm50.0$	$291.4^{abcA}\pm78.9$	0.4983	$235.7^{bcB}\pm58.3$	$104.5^{aA}\pm39.3$	0.0320	$222.7^{deA}\pm25.1$	$180.8^{abcA}\pm26.9$	0.1200
Rib-eye 2	$16.7^{abcA}\pm4.6$	$28.0^{abA}\pm7.4$	0.0896	$77.4^{aB}\pm20.9$	$25.7^{aA}\pm7.6$	0.0158	$418.5^{bcA}\pm172.8$	$524.3^{cA}\pm173.3$	0.4957	$403.3^{cA}\pm177.7$	$510.4^{bA}\pm294.9$	0.3186	$276.2^{dA}\pm39.1$	$406.8^{eA}\pm71.5$	0.0501
Rib-eye 3	$5.7^{abA}\pm0.9$	$58.5^{abcdB}\pm15.2$	0.0039	$14.5^{aA}\pm4.7$	$129.6^{aB}\pm38.3$	0.0066	$360.7^{abcA}\pm77.1$	$322.2^{abcA}\pm76.4$	0.5722	$123.4^{abA}\pm38.8$	$66.9^{aA}\pm31.6$	0.1223	$299.2^{dA}\pm34.0$	$244.2^{bcdA}\pm55.0$	0.2141
Rib-eye 4	$36.0^{abcdA}\pm5.9$	$40.4^{abcA}\pm7.0$	0.4546	$841.7^{bcdA}\pm196.6$	$554.1^{abcA}\pm141.5$	0.1088	$159.6^{aA}\pm40.5$	$170.5^{aA}\pm48.0$	0.7785	$109.2^{abA}\pm48.7$	$117.3^{aA}\pm66.2$	0.8727	$67.5^{aA}\pm7.6$	$71.8^{aA}\pm8.7$	0.5507
Rib-eye 5	$66.9^{cdA}\pm7.1$	$85.8^{bcdeA}\pm20.7$	0.2095	$843.5^{bcdA}\pm151.6$	$868.4^{bcdA}\pm480.3$	0.9359	$171.0^{abA}\pm34.0$	$215.8^{abA}\pm84.4$	0.4418	$57.4^{abA}\pm30.2$	$53.4^{aA}\pm28.4$	0.8755	$112.1^{abcA}\pm10.6$	$152.0^{abA}\pm35.5$	0.1358
Rib-eye 6	$1.9^{aA}\pm0.3$	$9.7^{aB}\pm4.0$	0.0276	$72.8^{aA}\pm22.6$	$96.7^{aA}\pm55.9$	0.5303	$113.5^{aA}\pm28.0$	$178.2^{aB}\pm24.7$	0.0398	$106.3^{abA}\pm44.8$	$75.5^{aA}\pm29.3$	0.3755	$96.7^{abcA}\pm18.1$	$137.4^{abB}\pm13.2$	0.0346
Rib-eye 7	$50.7^{abcdA}\pm7.2$	$68.0^{abcdeA}\pm16.2$	0.1667	$781.1^{bcdA}\pm152.8$	$841.6^{bcdA}\pm175.6$	0.6759	$162.0^{abA}\pm43.5$	$210.3^{abA}\pm80.8$	0.4134	$41.8^{aA}\pm10.3$	$30.8^{aA}\pm14.7$	0.3485	$78.4^{abA}\pm7.5$	$124.6^{abB}\pm11.3$	0.0041
Rib-eye 8	$159.7^{fA}\pm27.1$	$190.2^{fA}\pm44.5$	0.3678	$485.4^{abA}\pm156.4$	$476.1^{abcA}\pm95.5$	0.9341	$444.6^{cA}\pm67.1$	$485.9^{bcA}\pm165.4$	0.7091	$119.1^{abA}\pm41.2$	$111.8^{aA}\pm31.6$	0.8196	$264.8^{dA}\pm30$	$335.4^{deA}\pm73.0$	0.1966
Rib-eye 9	$49.4^{abcdA}\pm16.1$	$76.9^{abcdeA}\pm32.0$	0.2544	$1157.8^{cdA}\pm482.1$	$1463.1^{dA}\pm410.6$	0.4506	$213.1^{abcA}\pm57.4$	$201.9^{abA}\pm71.3$	0.8424	$83.9^{abA}\pm40.4$	$78.0^{aA}\pm30.7$	0.8503	$100.5^{abcA}\pm12.2$	$95.5^{aA}\pm10.8$	0.6195
Rib-eye 10	$54.4^{abcdA}\pm11.6$	$113.9^{deB}\pm13.7$	0.0045	$274.0^{abA}\pm140.9$	$161.1^{aA}\pm64.4$	0.2753	$284.4^{abcA}\pm57.1$	$345.3^{abcA}\pm87.6$	0.3699	$168.9^{abA}\pm56.8$	$165.6^{aA}\pm72.2$	0.9533	$145.3^{bcA}\pm22.9$	$221.8^{bcdB}\pm27.1$	0.0202
Rib-eye 11	$124.2^{efA}\pm42.6$	$99.8^{cdeA}\pm11.3$	0.3916	$444.3^{abA}\pm209.5$	$342.1^{abA}\pm77.5$	0.4725	$369.0^{abcA}\pm156.2$	$304.7^{abcA}\pm90.2$	0.5704	$118.4^{abA}\pm32.4$	$75.7^{aA}\pm41.9$	0.2349	$408.6^{eA}\pm46.4$	$291.1^{cdeA}\pm58.8$	0.0531
Sirloin 1	$57.5^{bcdA}\pm11.3$	$89.9^{bcdeA}\pm36.7$	0.2180	$615.1^{abcA}\pm167.0$	$488.4^{abcA}\pm191.1$	0.4360	$300.7^{abcA}\pm130.9$	$316.1^{abcA}\pm78.8$	0.8699	$88.5^{abA}\pm29.8$	$85.7^{aA}\pm56.5$	0.9432	$164.2^{cdA}\pm28.1$	$167.5^{abA}\pm20.3$	0.8787
Sirloin 2	$78.1^{deA}\pm26.6$	$133.5^{efA}\pm31.2$	0.0791	$888.6^{bcdA}\pm215.5$	$664.8^{abcA}\pm218.7$	0.2753	$259.1^{abcA}\pm67.5$	$344.2^{abcA}\pm139.1$	0.3943	$113.4^{abA}\pm91.4$	$118.9^{aA}\pm20.1$	0.9237	$153.9^{bcdA}\pm26.4$	$232.1^{bcdA}\pm54.1$	0.0875
Sirloin 3	$82.1^{deA}\pm22.0$	$93.5^{bcdeA}\pm24.1$	0.5775	$1277.6^{dA}\pm374.0$	$1057.3^{cdA}\pm377.6$	0.5125	$203.1^{abcA}\pm58.2$	$236.2^{abcA}\pm70.7$	0.5654	$73.5^{abB}\pm10.8$	$42.0^{aA}\pm9.7$	0.0198	$112.4^{abcA}\pm11.4$	$145.8^{abA}\pm19.8$	0.0649
p – value	<0.0001	<0.0001		<0.0001	<0.0001		0.0005	0.0025		<0.0001	<0.0001		<0.0001	<0.0001	

All data are presented as mean ± standard deviation (SD); different lowercase letters (a–e) within the same column indicate that mean values are significantly different (Tukey’s HSD, p ≤ 0.05), p - values obtained using one-way ANOVA; different uppercase letters (A–B) within the same row indicate significant differences between crust and interior (Student's t-test, p ≤ 0.05).

Table S4. Free amino acids profile of the crust and interior of commercial dry-aged beef steaks from Poland (not included in Table 8).

Steak type	Glutamic acid [mg/kg]			Threonine [mg/kg]			Glycine [mg/kg]			Alanine [mg/kg]			Tryptophan [mg/kg]		
	crust	interior	p – value (crust vs. interior)	crust	interior	p – value (crust vs. interior)	crust	interior	p – value (crust vs. interior)	crust	interior	p – value (crust vs. interior)	crust	interior	p – value (crust vs. interior)
Rib-eye 1	863.2^{deA}±175.0	586.1^{bcdA}±93.5	0.0728	285.0d^{eB}±12.6	234.2^{bcA}±20.2	0.0209	228.3^{abcA}±54.0	195.0^{abA}±39.1	0.4360	986.6^{abA}±200.0	731.4^{aA}±123.1	0.1328	53.5^{fgB}±4.8	31.2^{cA}±3.6	0.0030
Rib-eye 2	1485.8^{fA}±270.9	1416.4^{eA}±309.7	0.7846	489.0^{gA}±42.4	603.8^{eB}±35.2	0.0225	346.6^{cA}±72.0	394.7^{cA}±108.0	0.5560	1740.6^{cA}±406.2	1716.0^{bA}±377.1	0.9425	53.0^{efgA}±6.0	76.4^{fB}±8.7	0.0183
Rib-eye 3	938.8^{eA}±138.2	692.8^{cdA}±172.0	0.1257	412.9^{fgB}±37.7	274.0^{cA}±22.5	0.0054	293.6^{bA}±75.9	215.8^{abA}±59.1	0.2337	1386.5^{bcA}±449.5	882.7^{aA}±198.9	0.1505	72.9^{hA}±14.2	55.2^{eA}±5.8	0.1158
Rib-eye 4	308.9^{abcB}±63.7	172.5^{aA}±35.6	0.0317	160.1^{abA}±22.1	159.2^{aA}±20.5	0.9611	158.8^{abA}±40.9	141.6^{aA}±24.8	0.5672	561.0^{aA}±199.2	552.1^{aA}±81.3	0.9460	7.7^{abA}±1.2	10.9^{abA}±2.2	0.0947
Rib-eye 5	290.8^{abcA}±60.8	364.1^{abcA}±67.1	0.2333	144.0^{abA}±11.5	176.0^{abA}±32.8	0.1859	118.1^{aA}±30.2	136.8^{aA}±39.2	0.5486	530.5^{aA}±80.6	565.6^{aA}±79.8	0.6203	20.1^{abcdA}±2.8	30.1^{cB}±3.0	0.0134
Rib-eye 6	550.3^{bcdA}±128.4	564.0^{bcdA}±93.1	0.8883	162.1^{abcA}±20.1	165.7^{abA}±8.7	0.7904	165.9^{abA}±40.5	181.7^{abA}±47.7	0.6842	581.7^{aA}±79.0	527.3^{aA}±133.7	0.5766	2.5^{aA}±0.3	9.7^{abB}±1.5	0.0011
Rib-eye 7	209.8^{abA}±43.9	246.7^{abA}±50.7	0.3941	108.6^{aA}±6.7	147.1^{aB}±20.0	0.0341	153.0^{abA}±33.2	181.0^{abA}±45.9	0.4404	476.1^{aA}±129.3	596.0^{aA}±170.1	0.3861	16.8^{abcA}±2.3	28.2^{cB}±4.6	0.0184
Rib-eye 8	521.8^{abcdA}±102.3	592.1^{bcdA}±78.7	0.3987	383.6^{fA}±18.2	413.0^{dA}±27.6	0.1987	297.2^{bcA}±60.2	311.0^{bcA}±56.7	0.7869	1071.7^{abcA}±277.0	1027.8^{aA}±173.2	0.8275	76.9^{hA}±8.7	86.5^{fA}±10.0	0.2784
Rib-eye 9	460.1^{abcA}±109.7	298.2^{abA}±67.3	0.0949	161.5^{abcA}±15.4	148.3^{aA}±29.0	0.5241	207.3^{abcA}±37.7	194.7^{abA}±40.5	0.7135	952.8^{abA}±171.3	783.3^{aA}±142.8	0.2582	22.6^{bcdA}±2.2	20.7^{bcA}±3.3	0.4478
Rib-eye 10	975.1^{eA}±179.0	743.2^{dA}±152.6	0.1628	247.9^{cdA}±32.0	294.7^{cA}±29.6	0.1366	224.2^{abcA}±64.3	230.2^{abcA}±53.1	0.9068	1038.3^{abA}±281.1	889.0^{aA}±267.3	0.5414	35.2^{deA}±6.6	49.9^{deB}±5.9	0.0449
Rib-eye 11	541.2^{abcdA}±89.3	409.6^{abcdA}±63.0	0.1052	354.4^{efB}±51.5	260.0^{cA}±18.4	0.0403	194.4^{abcA}±53.1	153.8^{abA}±37.2	0.3390	1134.2^{abcA}±200.6	799.6^{aA}±202.8	0.1120	67.5^{ghA}±7.5	56.4^{eA}±5.0	0.0989
Sirloin 1	598.6^{cdeA}±103.9	735.8^{dA}±116.2	0.2020	276.2^{deA}±49.1	273.9^{cA}±18.5	0.9431	263.7^{abcA}±46.1	256.2^{abcA}±53.5	0.8629	824.7^{abA}±141.7	773.6^{aA}±215.1	0.7484	2.5^{aA}±0.4	2.5^{aA}±0.2	1.0000
Sirloin 2	528.6^{abcdA}±96.0	508.8^{abcdA}±84.5	0.8018	221.2^{bcdA}±26.7	297.0^{cB}±21.5	0.0185	195.5^{abcA}±60.1	242.6^{abcA}±74.5	0.4421	795.6^{abA}±110.4	892.0^{aA}±179.1	0.4718	37.7^{defA}±4.3	59.1^{eB}±8.8	0.0192
Sirloin 3	156.7^{aA}±24.5	273.2^{abB}±37.9	0.0110	161.0^{abcA}±15.4	187.9^{abA}±13.3	0.0837	177.2^{abA}±34.4	197.1^{abA}±53.2	0.6151	695.7^{abA}±151.0	667.4^{aA}±170.7	0.8401	28.5^{cdA}±5.1	36.1^{cdA}±6.2	0.1759
p – value	<0.0001	<0.0001		<0.0001	<0.0001		0.0003	0.0003		<0.0001	<0.0001		<0.0001	<0.0001	

All data are presented as mean ± standard deviation (SD); different lowercase letters (a–h) within the same column indicate that mean values are significantly different (Tukey's HSD, p ≤ 0.05), p - values obtained using one-way ANOVA; different uppercase letters (A–B) within the same row indicate significant differences between crust and interior (Student's t-test, p ≤ 0.05).

Table S5. Free amino acids profile of the crust and interior of commercial dry-aged beef steaks from Poland (not included in Table 8).

Steak type	Proline [mg/kg]			Valine [mg/kg]			Methionine [mg/kg]			Leucine and isoleucine [mg/kg]			Arginine [mg/kg]		
	crust	interior	p – value (crust vs. interior)	crust	interior	p – value (crust vs. interior)	crust	interior	p – value (crust vs. interior)	crust	interior	p – value (crust vs. interior)	crust	interior	p – value (crust vs. interior)
Rib-eye 1	209.1^{cdeA}±46.0	138.1^{abA}±13.2	0.0621	325.8^{cdA}±34.6	265.3^{abcdA}±45.4	0.1402	135.0^{cdA}±39.3	112.5^{abcA}±28.8	0.4682	668.4^{bcdA}±124.0	493.9^{abcA}±64.8	0.0969	34.4^{abA}±13.8	13.4^{Aa}±1.8	0.0596
Rib-eye 2	389.9^{gA}±50.6	349.3^{cA}±59.6	0.4185	515.3^{eA}±86.2	643.2^{eA}±78.1	0.1298	172.7^{deA}±24.0	284.2^{eB}±44.7	0.0191	752.7^{cdA}±72.3	1204.0^{eA}±356.8	0.0983	26.2^{aA}±7.7	20.6^{aA}±4.2	0.3297
Rib-eye 3	219.4^{defB}±26.6	126.8^{abA}±38.2	0.0263	426.3^{deB}±42.8	311.4^{bcdA}±30.6	0.0194	211.1^{eA}±45.8	150.4^{bcdA}±36.3	0.1463	898.1^{deA}±209.4	619.8^{bcdA}±154.8	0.1377	26.9^{abB}±4.0	13.2^{aA}±2.8	0.0081
Rib-eye 4	129.8^{abcdA}±26.0	126.9^{abA}±50.6	0.9332	117.0^{aA}±16.2	125.9^{aA}±14.3	0.5173	39.0^{aA}±3.2	43.8^{aA}±6.3	0.3022	194.0^{aA}±36.1	205.7^{aA}±23.5	0.6604	13.2^{aA}±4.6	13.8^{aA}±1.6	0.8414
Rib-eye 5	79.7^{aA}±11.1	89.5^{aA}±16.0	0.4311	152.0^{abA}±15.4	203.4^{abcB}±22.2	0.0298	66.6^{abcA}±4.6	91.7^{abA}±19.6	0.0977	287.7^{aA}±49.8	386.8^{abA}±111.9	0.2233	157.5^{cA}±54.9	224.2^{bcA}±43.9	0.1754
Rib-eye 6	110.4^{abcdA}±23.6	74.3^{aA}±13.6	0.0835	169.9^{abA}±23.1	190.9^{abA}±21.2	0.3116	59.0^{abA}±6.7	76.5^{abA}±13.9	0.1207	260.2^{aA}±61.0	336.0^{abA}±86.0	0.2813	10.1^{aA}±2.5	14.8^{aA}±3.8	0.1471
Rib-eye 7	75.8^{aA}±14.6	77.9^{aA}±15.0	0.8737	116.2^{aA}±14.2	163.4^{aA}±27.8	0.0590	49.4^{abA}±8.6	81.6^{abA}±19.7	0.0603	215.0^{aA}±48.5	331.1^{abA}±106.4	0.1607	111.8^{abcA}±21.3	199.2^{bcB}±32.7	0.0178
Rib-eye 8	247.4^{efA}±40.8	218.8^{bA}±37.4	0.4218	454.9^{deA}±91.9	514.4^{eA}±66.9	0.4157	171.4^{deA}±25.8	208.8^{deA}±17.2	0.1047	762.8^{cdA}±79.9	926.2^{deA}±129.6	0.1367	370.4^{dA}±106.2	526.9^{dA}±74.8	0.1052
Rib-eye 9	98.6^{abcA}±8.9	79.6^{aA}±16.8	0.1592	140.3^{abA}±14.9	132.1^{aA}±20.3	0.6029	61.4^{abA}±11.8	61.0^{aA}±11.4	0.9668	277.8^{A}±32.9	259.4^{abA}±56.5	0.6512	94.9^{abcA}±19.5	143.1^{bA}±29.5	0.0773
Rib-eye 10	169.3^{abcdeA}±30.4	163.5^{abA}±32.0	0.8319	234.5^{abcA}±22.5	321.9^{bcdA}±72.5	0.1167	97.7^{abcA}±34.2	141.3^{bcdA}±38.3	0.2150	430.0^{abA}±113.2	613.8^{bcdA}±93.3	0.0959	49.3^{abcA}±16.1	167.8^{bB}±58.6	0.0279
Rib-eye 11	326.7^{fgB}±49.6	134.6^{abA}±21.5	0.0035	475.3^{eA}±71.4	361.4^{gA}±33.0	0.0663	210.7^{eA}±30.6	175.2^{cdA}±10.8	0.1311	1101.7^{eA}±193.6	792.6^{cdA}±128.5	0.0826	287.6^{dA}±69.1	305.3^{cA}±68.0	0.7676
Sirloin 1	195.5^{bcdeA}±83.8	166.6^{abA}±41.7	0.6212	264.0^{bcA}±37.0	257.2^{abcdA}±60.1	0.8761	117.0^{bcdA}±13.5	117.0^{abcA}±36.3	0.9992	496.5^{abcA}±84.5	486.3^{abcA}±107.4	0.9039	11.1^{aA}±3.4	8.8^{aA}±2.7	0.4135
Sirloin 2	133.1^{abcdA}±25.0	159.5^{abA}±56.8	0.5038	220.3^{abcA}±48.0	342.4^{cdA}±80.0	0.0860	99.9^{abcA}±10.4	149.5^{bcdB}±21.2	0.0222	426.7^{abA}±81.8	634.1^{bcdA}±104.3	0.0535	13.1^{aA}±2.6	111.1^{abB}±12.2	0.0001
Sirloin 3	95.5^{abA}±15.6	100.8^{aA}±24.4	0.7674	156.9^{abA}±31.7	195.3^{abA}±17.3	0.1397	69.1^{abcA}±6.0	92.5^{abB}±10.5	0.0286	286.7^{aA}±89.5	371.6^{abA}±48.3	0.2218	146.8^{bcA}±47.9	210.5^{bcA}±53.9	0.2008
p – value	<0.0001	<0.0001		<0.0001	<0.0001		<0.0001	<0.0001		<0.0001	<0.0001		<0.0001	<0.0001	

All data are presented as mean ± standard deviation (SD); different lowercase letters (a–e) within the same column indicate that mean values are significantly different (Tukey's HSD, $p \leq 0.05$), p - values obtained using one-way ANOVA; different uppercase letters (A–B) within the same row indicate significant differences between crust and interior (Student's t-test, $p \leq 0.05$).

Table S6. Biogenic amines (BAs) profile of the crust and interior of commercial dry-aged beef steaks from Poland (not included in Table 9).

Steak type	Agmatine (AGM) [mg/kg]			2-phenylethylamine (PEA) [mg/kg]			Tryptamine (TRM) [mg/kg]			Spermine (SPM) [mg/kg]			Spermidine (SPD) [mg/kg]		
	**crust**	**interior**	**p – value (crust vs. interior)**	**crust**	**interior**	**p – value (crust vs. interior)**	**crust**	**interior**	**p – value (crust vs. interior)**	**crust**	**interior**	**p – value (crust vs. interior)**	**crust**	**interior**	**p – value (crust vs. interior)**
Rib-eye 1	$16.9^{bcA}\pm6.1$	$12.7^{cdA}\pm4.1$	0.3804	$0.6^{aA}\pm0.2$	$0.3^{aA}\pm0.1$	0.0670	$13.5^{abcB}\pm2.4$	$6.3^{abA}\pm0.9$	0.0081	$36.5^{abA}\pm9.9$	$42.9^{aA}\pm9.3$	0.4616	$3.1^{abA}\pm1.1$	$1.5^{aA}\pm0.4$	0.0857
Rib-eye 2	$7.1^{abA}\pm2.4$	$4.2^{bA}\pm1.6$	0.1520	$7.9^{bA}\pm2.9$	$4.3^{bA}\pm0.9$	0.1117	$25.6^{cA}\pm4.9$	$19.2^{cA}\pm5.5$	0.2096	$48.0^{abA}\pm10.1$	$41.0^{aA}\pm5.0$	0.3413	$8.9^{dA}\pm2.2$	$4.4^{abA}\pm2.1$	0.0627
Rib-eye 3	$0.3^{aB}\pm0.1$	$0.2^{aA}\pm0.1$	0.0253	$0.3^{aA}\pm0.1$	$0.3^{aA}\pm0.1$	1.0000	$11.2^{abB}\pm2.5$	$4.9^{abA}\pm1.5$	0.0202	$37.3^{abA}\pm4.5$	$39.0^{aA}\pm6.5$	0.7284	$1.8^{aA}\pm0.5$	$1.0^{aA}\pm0.4$	0.0906
Rib-eye 4	$0.2^{aA}\pm0.1$	$0.1^{aA}\pm0.1$	1.0000	$0.3^{aA}\pm0.1$	$0.3^{aA}\pm0.1$	1.0000	$6.9^{aA}\pm1.5$	$6.2^{abA}\pm2.6$	0.7104	$60.6^{bA}\pm17.1$	$57.0^{aA}\pm6.3$	0.7495	$8.0^{cdA}\pm2.0$	$6.3^{aA}\pm2.3$	0.3923
Rib-eye 5	$2.5^{aA}\pm0.8$	$1.6^{aA}\pm0.6$	0.2088	$0.3^{aA}\pm0.1$	$0.3^{aA}\pm0.1$	1.0000	$2.0^{aA}\pm0.4$	$2.0^{aA}\pm0.4$	1.0000	$49.6^{abA}\pm7.1$	$51.3^{aA}\pm6.7$	0.7777	$3.6^{abA}\pm0.9$	$3.2^{abA}\pm1.1$	0.6495
Rib-eye 6	$49.0^{cB}\pm19.2$	$0.3^{aA}\pm0.1$	0.0117	$1.6^{aB}\pm0.5$	$0.3^{aA}\pm0.1$	0.0095	$23.5^{cA}\pm7.1$	$17.0b^{cA}\pm5.8$	0.2875	$51.2^{abA}\pm7.2$	$51.2^{aA}\pm13.9$	1.0000	$3.7^{abA}\pm1.6$	$3.4^{abA}\pm1.0$	0.7932
Rib-eye 7	$26.4^{bcA}\pm8.3$	$17.3^{cdA}\pm7.2$	0.2251	$0.3^{aA}\pm0.1$	$0.3^{aA}\pm0.1$	1.0000	$2.0^{aA}\pm0.3$	$2.0^{aA}\pm0.5$	1.0000	$47.0^{abA}\pm10.2$	$41.6^{aA}\pm8.9$	0.5286	$7.1^{bcdA}\pm2.8$	$3.3^{abA}\pm0.8$	0.0860
Rib-eye 8	$12.7^{abA}\pm4.4$	$7.6^{bA}\pm2.1$	0.1410	$0.3^{aA}\pm0.1$	$0.3^{aA}\pm0.1$	1.0000	$2.0^{aA}\pm0.6$	$2.0^{aA}\pm0.4$	1.0000	$44.8^{abA}\pm7.7$	$46.3^{aA}\pm13.3$	0.8740	$2.0^{aA}\pm0.8$	$1.9^{aA}\pm0.7$	0.8774
Rib-eye 9	$42.6^{cA}\pm17.2$	$28.0^{dA}\pm12.6$	0.3012	$0.3^{aA}\pm0.1$	$0.3^{aA}\pm0.1$	1.0000	$2.0^{aA}\pm0.3$	$2.0^{aA}\pm0.4$	1.0000	$55.4^{bA}\pm10.4$	$46.0^{aA}\pm11.5$	0.3533	$4.4^{abcA}\pm1.1$	$3.4^{abA}\pm1.1$	0.3226
Rib-eye 10	$8.7^{abB}\pm4.4$	$1.5^{abA}\pm0.4$	0.0491	$0.3^{aA}\pm0.1$	$0.3^{aA}\pm0.1$	1.0000	$2.0^{aA}\pm0.6$	$2.0^{aA}\pm0.5$	1.0000	$49.5^{abA}\pm6.5$	$41.1^{aA}\pm10.2$	0.2967	$4.0^{abcA}\pm0.9$	$2.2^{aA}\pm0.8$	0.0641
Rib-eye 11	$0.2^{aA}\pm0.1$	$0.1^{aA}\pm0.1$	1.0000	$0.3^{aA}\pm0.1$	$0.3^{aA}\pm0.1$	1.0000	$2.0^{aA}\pm0.5$	$2.0^{aA}\pm0.4$	1.0000	$24.7^{aA}\pm6.0$	$34.6^{aA}\pm7.6$	0.1529	$1.7^{aA}\pm0.6$	$1.0^{aA}\pm0.4$	0.1665
Sirloin 1	$0.4^{aA}\pm0.1$	$0.3^{aA}\pm0.1$	0.3170	$2.1^{aB}\pm0.5$	$1.0^{aA}\pm0.4$	0.0331	$47.8^{dA}\pm7.5$	$47.7^{dA}\pm16.4$	0.9928	$39.4^{abA}\pm9.8$	$41.9^{aA}\pm9.8$	0.7700	$3.3^{abA}\pm1.4$	$2.9^{abA}\pm1.1$	0.7175
Sirloin 2	$10.6^{abB}\pm5.4$	$0.1^{aA}\pm0.1$	0.0277	$0.3^{aA}\pm0.1$	$0.3^{aA}\pm0.1$	1.0000	$2.0^{aA}\pm0.4$	$2.0^{aA}\pm0.6$	1.0000	$38.6^{abA}\pm4.4$	$39.8^{aA}\pm5.4$	0.7810	$2.5^{aA}\pm1.0$	$1.9^{aA}\pm0.9$	0.4730
Sirloin 3	$18.0^{bcB}\pm4.9$	$2.7a^{bA}\pm0.7$	0.0057	$0.3^{aA}\pm0.1$	$0.3^{aA}\pm0.1$	1.0000	$2.0^{aA}\pm0.6$	$2.0^{aA}\pm0.4$	1.0000	$43.1^{abA}\pm9.1$	$42.3^{aA}\pm7.3$	0.9116	$2.1^{aA}\pm0.6$	$1.8^{aA}\pm0.7$	0.5801
p – value	<0.0001	<0.0001		<0.0001	<0.0001		<0.0001	<0.0001		0.0080	0.3045		<0.0001	0.0005	

All data are presented as mean ± standard deviation (SD); different lowercase letters (a–d) within the same column indicate that mean values are significantly different (Tukey's HSD, $p \le 0.05$), p - values obtained using one-way ANOVA; different uppercase letters (A–B) within the same row indicate significant differences between crust and interior (Student's t-test, $p \le 0.05$).

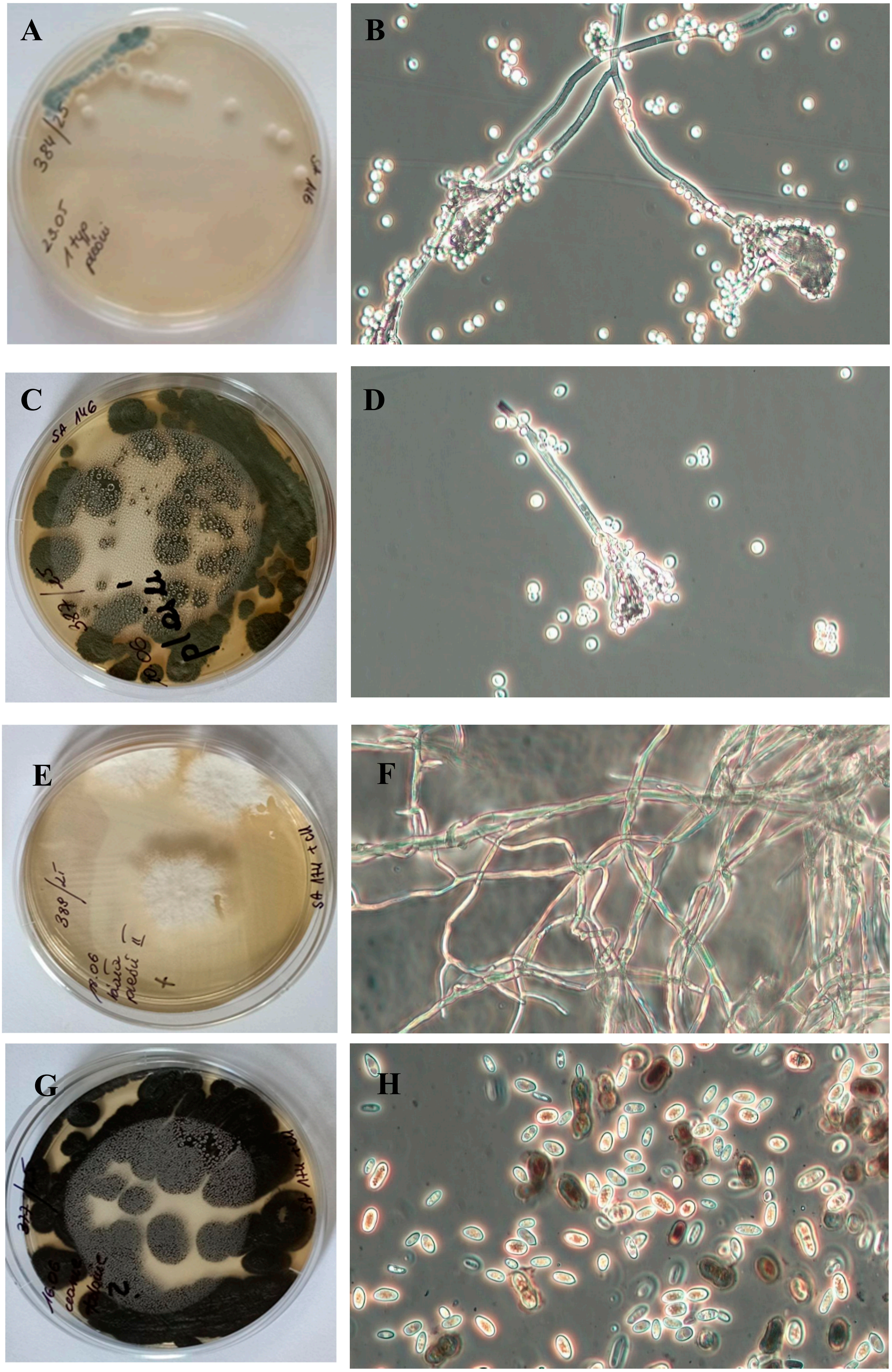
A
B
C
D
E
F
G
H

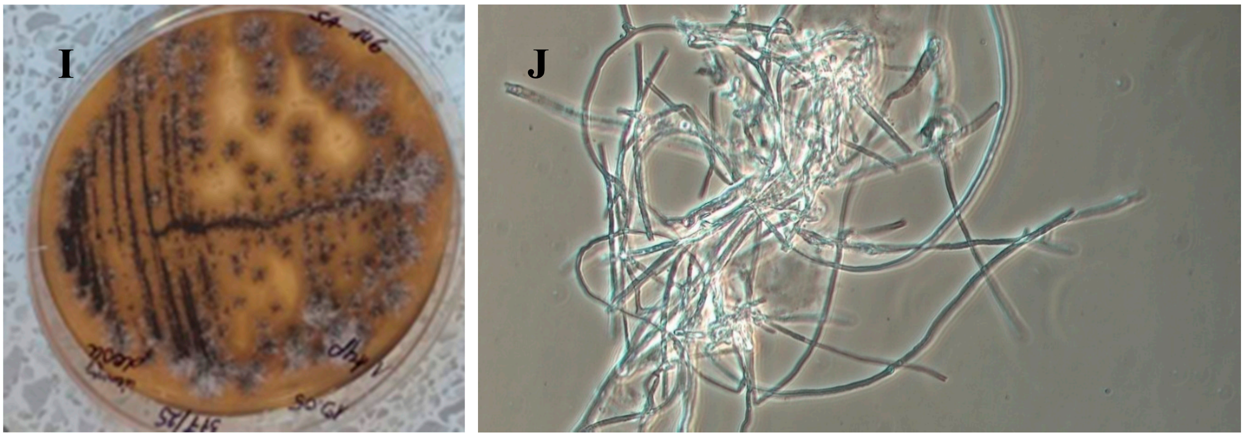

Figure S1. Morphological and microscopic characteristics of mold isolates from dry-aged beef. Colony appearance (A) and microscope image (B) of *Penicillium palitans*; colony appearance (C) and microscope image (D) of *Penicillium solitum*; colony appearance (E) and microscope image (F) of *Iprex laceratus*; colony appearance (G) and microscope image (H) of *Aureobasidium melanogenum*; colony appearance (I) and microscope image (J) of *Phoma herbarum*.
